# The chromatin remodeling enzyme Chd4 regulates genome architecture in the mouse brain

**DOI:** 10.1038/s41467-020-17065-z

**Published:** 2020-07-09

**Authors:** Jared V. Goodman, Tomoko Yamada, Yue Yang, Lingchun Kong, Dennis Y. Wu, Guoyan Zhao, Harrison W. Gabel, Azad Bonni

**Affiliations:** 1 Department of Neuroscience, Washington University School of Medicine, St. Louis, MO USA; 2 Medical Scientist Training Program, Washington University School of Medicine, St. Louis, MO USA; 3 Faculty of Medicine, University of Tsukuba, Tsukuba, Ibaraki Japan; 4 Department of Neurobiology, Northwestern University, Evanston, IL USA

**Keywords:** Developmental biology, Gene regulation, Chromatin remodelling, Chromatin structure, Neuroscience

## Abstract

The development and function of the brain require tight control of gene expression. Genome architecture is thought to play a critical regulatory role in gene expression, but the mechanisms governing genome architecture in the brain in vivo remain poorly understood. Here, we report that conditional knockout of the chromatin remodeling enzyme Chd4 in granule neurons of the mouse cerebellum increases accessibility of gene regulatory sites genome-wide in vivo. Conditional knockout of Chd4 promotes recruitment of the architectural protein complex cohesin preferentially to gene enhancers in granule neurons in vivo. Importantly, in vivo profiling of genome architecture reveals that conditional knockout of Chd4 strengthens interactions among developmentally repressed contact domains as well as genomic loops in a manner that tightly correlates with increased accessibility, enhancer activity, and cohesin occupancy at these sites. Collectively, our findings define a role for chromatin remodeling in the control of genome architecture organization in the mammalian brain.

## Abstract (web summary)

The mechanisms underlying gene regulation and genome architecture remain poorly understood. Here, the authors investigate the role of chromatin remodelling enzyme Chd4 in granule neurons of the mouse cerebellum and find that conditional knockout of Chd4 preferentially activates enhancers and modulates genome architecture at a genome-wide level.

## Introduction

Precise control of gene expression is required for the establishment and refinement of neural circuits^1,2^. Regulation of chromatin organization through DNA methylation, post-translational modifications of histone proteins, and nucleosome remodeling represents a fundamental facet of gene expression control^3–6^. Among these mechanisms, nucleosome remodeling, which comprises changes in nucleosome spacing, density, or subunit composition, remains perhaps the most poorly understood^7,8^.

Chromatin remodeling enzymes, which mediate nucleosome remodeling, have been of wide interest. Mutations of chromatin remodeling enzymes often cause neurodevelopmental disorders of cognition including autism spectrum disorders and intellectual disability^9^, suggesting a critical role for these proteins in neuronal connectivity and plasticity. Recent studies have highlighted crucial roles for the chromatin remodeling enzyme Chd4, also mutated in syndromic intellectual disability^10–12^, in the development and plasticity of the brain^9^. Depletion of Chd4, a core member of the nucleosome remodeling and deacetylase (NuRD) complex^13–15^, disrupts neuronal connectivity in mice^16–18^. Consequently, conditional knockout of Chd4 in cerebellar granule neurons impairs sensorimotor neural coding and cerebellar-dependent learning^18^. At a cellular level, Chd4 drives granule neuron/Purkinje cell synapse formation and the maturation of granule neuron dendrites via distinct mechanisms^16,18^. At a molecular level, Chd4 decommissions the promoters of developmentally regulated genes via alterations of histone tail modifications and thereby drives granule neuron/Purkinje cell synapse formation^16^. By contrast, Chd4 triggers deposition of the histone variant H2A.z at promoters of neuronal activity genes, leading to acute shutoff of activity genes and consequent pruning of granule neuron dendrites^18^. Importantly, in addition to binding gene promoters, Chd4 binds widely to enhancer regulatory elements in the brain^18^. Because enhancers play crucial roles in the regulation of gene expression and genome biology^19^, the finding that Chd4 occupies gene enhancers raises the fundamental question on Chd4 function and mechanisms in the regulation of enhancers in the brain. However, the role of Chd4 in the control of gene enhancers in the brain remains poorly understood.

In recent years, three-dimensional genome architecture has been recognized to robustly influence spatially the regulatory effects of enhancers on gene expression. Genome architecture features several elements including the local enrichment of contacts across a contiguous genomic region into contact domains or topologically associating domains (TADs) and the coalescence of non-contiguous genomic regions into loops^6,20,21^. Loop domains may form upon extrusion of DNA by the protein complex cohesin up to loop anchor points. The transcription factor Ctcf often occupies boundaries of loop domains, preventing contacts across loop boundaries^6,22–24^. Importantly, loops also bring together other genomic regions such as gene promoters and enhancers^19^. In contrast, compartmental domains are devoid of loops at their boundaries and may form through homotypic interactions among genomic regions with similar epigenomic status^6^. The composite of these local interactions emerges as higher order structures termed compartments^6,25^.

Genome architecture is dynamic during neuronal differentiation^26^, suggesting regulation of genome architecture may play a critical role in brain development. Mutations of cohesin complex proteins cause syndromic intellectual disability^27^, further corroborating a key function for genome architecture regulation in brain development. However, the mechanisms that control genome architecture in the brain remain largely unexplored. In yeast, the Rsc chromatin remodeling complex interacts with cohesin and the cohesin loading complex^28^. In murine embryonic stem cells, the Iswi family remodeler Snf2h promotes Ctcf binding to the genome, and thus regulates formation of contact domains^29^. These studies raise the fundamental question of whether chromatin remodeling enzymes might participate in the organization of genome architecture in the brain.

Here, we uncover a function for the chromatin remodeling enzyme Chd4 in the organization of genome architecture in the mouse brain in vivo. Conditional knockout of Chd4 in granule neurons of the mouse cerebellum increases the accessibility of gene promoters and enhancers genome-wide in vivo. Remarkably, conditional knockout of Chd4 promotes recruitment of the architectural protein complex cohesin to gene enhancers in granule neurons in vivo. Importantly, analyses of genome architecture in vivo demonstrate that conditional knockout of Chd4 strengthens interactions among developmentally repressed contact domains as well as genomic loops, consistent with changes in the epigenetic and gene expression status of regions underlying these architectural features. In sum, our findings define a role for chromatin remodeling in the organization of genome architecture in the developing brain.

## Results

### Chd4 regulates genomic accessibility and cohesin binding

**Fig. 1:**
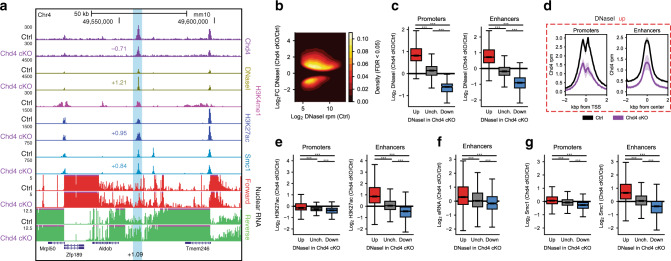
Chd4 preferentially modulates enhancer activation and cohesin binding. **a** Genome-browser snapshot of a region containing the *Aldob* gene locus on chromosome 4 displaying the ChIP-seq profiles of Chd4, H3K4me1, H3K27ac and Smc1 as well as DNaseI-seq and nuclear RNA-seq from the control and Chd4 cKO cerebellum. Light blue denotes an enhancer upstream of the *Aldob* gene. Numbers indicate the Log2 change in signal in the Chd4 cKO cerebellum, including that of eRNA. **b** MA density plot of DHS sites called as significant (FDR < 0.05) by DESeq2. **c** Boxplot of DnaseI change between the Chd4 cKO and control cerebellum at (left) promoters and (right) enhancers with increased (*n* = 19,389 promoters; *n* = 67,245 enhancers), unchanged (*n* = 15,141 promoters; *n* = 44,661 enhancers), and decreased (*n* = 828 promoters, *n* = 27,750 enhancers) accessibility. **d** Aggregate plot of Chd4 density in the control and Chd4 cKO cerebellum at (left) promoters and (right) enhancers with increased accessibility. Mean ± SEM. **e** Boxplot of H3K27ac change between the Chd4 cKO and control cerebellum at (left) promoters and (right) enhancers with increased (*n* = 19,389 promoters; *n* = 67,245 enhancers), unchanged (*n* = 15,141 promoters; *n* = 44,661 enhancers), and decreased (*n* = 828 promoters, *n* = 27,750 enhancers) accessibility. **f** Boxplot of eRNA change between the Chd4 cKO and control cerebellum at enhancers with increased (*n* = 2969), unchanged (*n* = 1455), and decreased (*n* = 526) accessibility. **g** Boxplot of Smc1 change between the Chd4 cKO and control cerebellum at (left) promoters and (right) enhancers with increased (*n* = 19,389 promoters; *n* = 67,245 enhancers), unchanged (*n* = 15,141 promoters; *n* = 44,661 enhancers), and decreased (*n* = 828 promoters, *n* = 27,750 enhancers) accessibility. *P*-values for all comparisons in this figure were calculated by the two-sided Kruskal–Wallis H-test for independent samples with Dunn’s post hoc T-test and corrected for multiple comparisons by the Bonferroni–Hochberg procedure. *p* < *0.001*.

To characterize the nucleosome remodeling activity of Chd4 in the brain, we assessed the effect of conditional knockout of Chd4 in granule neurons of the mouse cerebellum on genomic accessibility using DNaseI-hypersensitivity sequencing (DNaseI-seq)^16,18^. Because granule neurons outnumber all other neurons in the cerebellum^30^, they represent an ideal system for characterizing epigenetic and transcriptional mechanisms in the brain in vivo^9,16,18,31^. DNaseI-seq analyses from the cerebellum of postnatal day 22 (P22) control and conditional Chd4 knockout mice revealed a widespread increase in genomic accessibility upon Chd4 depletion (Fig. 1a–c, Supplementary Fig. 1A–C). Notably, accessibility was decreased at a minority of sites following Chd4 loss (Fig. 1b). Importantly, Chd4 occupied active promoters, marked by acetylated histone H3K27, and active enhancers, marked by acetylated histone H3K27 and monomethylated histone H3K4, which displayed increased accessibility upon conditional knockout of Chd4 (Fig. 1d, Supplementary Fig. 1A–D). Examples of such sites included the promoter of the *Zfp956* gene and enhancer downstream of the *Aldob* gene (Fig. 1a, Supplementary Fig. 1A, B)^16,18^. Chd4 protein remains expressed in the cerebellum of conditional Chd4 knockout mice within Purkinje neurons, inhibitory neurons, and a subset of granule neurons in which the Gabra6 promoter does not induce Cre expression^18^, likely explaining the residual Chd4 ChIP-seq signal in the cerebellum of conditional Chd4 knockout mice (Fig. 1d, Supplementary Fig. 1A–D). Corroborating our results of increased genomic accessibility upon Chd4 loss in granule neurons, recent data suggest Chd4 may reduce nucleosome accessibility in murine embryonic stem cells and immature B cells^32–35^. Taken together, these data demonstrate that Chd4 suppresses genomic accessibility in the mammalian brain.

We next characterized how the regulation of genomic accessibility by Chd4 might influence the activity state of promoters and enhancers in the mouse cerebellum in vivo. In analyses of chromatin immunoprecipitation followed by sequencing (ChIP-seq), using levels of H3K27 acetylation as a surrogate of regulatory site activity^16,36^, changes in promoter activity failed to correlate effectively with increased accessibility at these sites upon Chd4 loss in the cerebellum (Fig. 1e, Supplementary Fig. 1B,C, 1E). Strikingly, however, we found that enhancer activity increased robustly at sites of increased accessibility in the cerebellum upon Chd4 depletion (Fig. 1a, e, Supplementary Fig. 1E). We further assessed the effect of Chd4 on enhancer activity by measuring the transcriptional activity of enhancers in the brain. We performed total RNA-seq from the cell nucleus in the cerebellum from control and conditional Chd4 knockout mice. Analysis of enhancer RNAs (eRNAs) genome-wide revealed that the change in accessibility at enhancers correlated with changes in eRNA expression in the cerebellum from conditional Chd4 knockout mice (Fig. 1f). Taken together, these results suggest that Chd4 regulation of genomic accessibility selectively influences enhancer site activity in the brain.

Besides histone tail modifications, enhancers represent key sites for binding of DNA regulatory proteins including the ring-like genome architecture protein complex cohesin^19,37^. We asked whether Chd4 regulation of genomic accessibility might control cohesin binding to enhancers. In ChIP-seq analyses using an antibody to the cohesin complex protein Smc1, we found that Smc1 occupancy robustly increased at enhancer sites with increased accessibility in the cerebellum in conditional Chd4 knockout mice (Fig. 1a, g, Supplementary Fig. 1C, 1E). These data suggest that Chd4 regulates both accessibility and cohesin binding at enhancers in the brain.

### Chd4 regulates features of genome architecture in the brain

The finding that Chd4 strongly influences genomic accessibility, enhancer activity, and cohesin binding at enhancers led us to determine whether Chd4 regulates genome architecture in the brain. We therefore performed in situ chromosome conformation capture with high-throughput sequencing (Hi-C)^21^ in the cerebellum of control and conditional Chd4 knockout mice. We identified over 1.7 billion genomic contacts in these analyses from three biological replicates per condition to attain 6 kb resolution contact matrices (Supplementary Fig. 2A, B). Biological replicates were highly concordant, so all replicates were pooled for further analysis (Supplementary Fig. 2C). Sequencing to this depth revealed distinct features of genome-wide contacts including compartmentalization, contact domains, and loops in the cerebellum (Supplementary Fig. 2D, E).

To determine the role of Chd4 in the organization of contact domains, we first characterized these domains in the mouse cerebellum. Using the algorithm Arrowhead^21^, we identified 7,796 contact domains in the cerebellum of control and conditional Chd4 knockout mice (Supplementary Fig. 3A). To assess if Chd4 might distinctly affect loop and compartmental domains, we segregated these domains further into those harboring genomic loops at domain borders, i.e. loop domain, and those without border loops, i.e. compartmental domain. Using the algorithm HiCCUPS^21^, we identified 11,525 loops in the cerebellum of control and conditional Chd4 knockout mice (Supplementary Fig. 3B), demarcating 2,752 loop domains and 5,044 compartmental domains (Supplementary Fig. 3C–E).

**Fig. 2:**
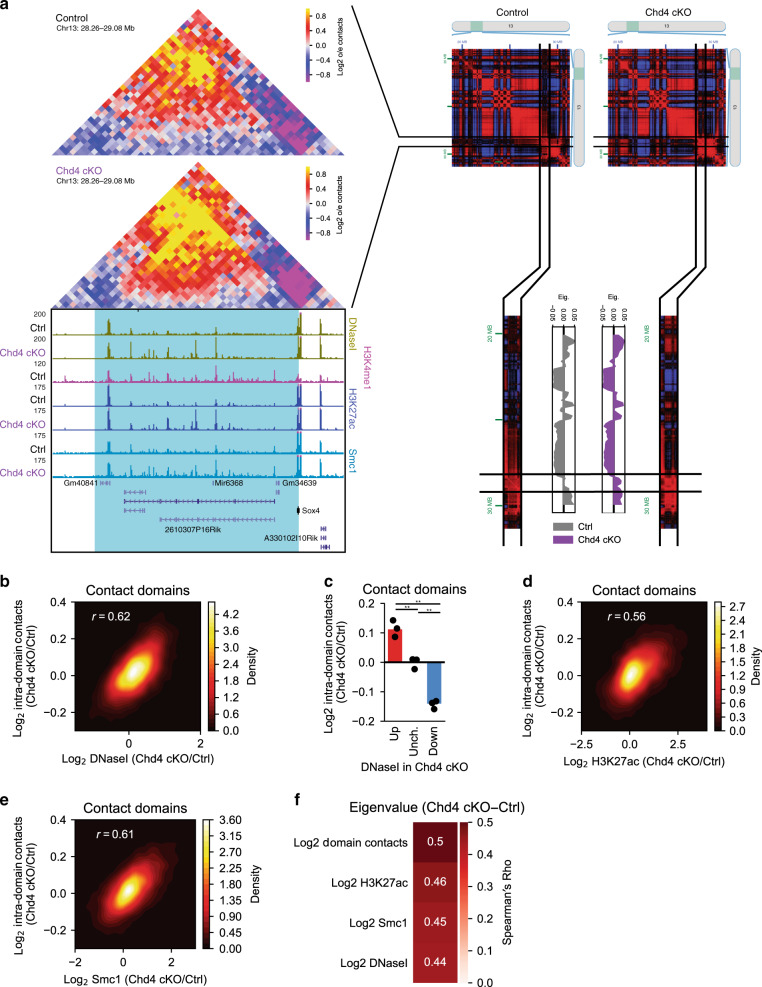
Chd4 alters contact domain interactions and compartmentalization. **a** (Left) Hi-C contact matrix of a contact domain on chromosome 13 and the flanking region. Below is a genome-browser snapshot of the region corresponding to the Hi-C contact matrix displaying the ChIP-seq profiles of H3K4me1, H3K27ac, and Smc1 as well as DNaseI-seq from the control and Chd4 cKO cerebellum. (Right) Juicebox browser snapshot of the Pearson’s correlation matrix of a region on chromosome 13 surrounding the contact domain. Below is the eigenvalue of the corresponding region on chromosome 13 in 150 kb bins, with the region surrounding the contact domain highlighted. **b** Density plot comparing the change in contact domain DNaseI-seq with the change in Hi-C contacts within the domain in the control and Chd4 cKO cerebellum. Pearson’s *r*, *p* < *0.001*. **c** Change in Hi-C contacts within domains with increased (Log_2_FC > 0.585), unchanged (−0.585 < Log_2_FC < 0.585), and decreased (−0.585 < Log_2_FC) accessibility in the Chd4 cKO cerebellum. Points represent average of domains in individual biological replicates (*n* = 3). One-way ANOVA with Tukey’s post hoc HSD test. **p < *0.01*. **d**, **e** Density plot comparing the change in contact domain (**e**) H3K27ac and (**f**) Smc1 ChIP-seq with the change in Hi-C contacts within the domain in the control and Chd4 cKO cerebellum. Pearson’s *r*, *p* < *0.001*. **f** Spearman’s Rho comparing the difference in eigenvalue among contact domains and the change in domain contacts or other epigenomic features (Chd4 cKO/Ctrl).

We next assessed the effect of conditional Chd4 knockout on contact domain interactions in conjunction with effects on genomic accessibility, H3K27 acetylation, and cohesin binding. Alterations of genomic interactions within contact domains correlated with changes in epigenomic features at enhancers within these domains. For example, interactions within a contact domain on chromosome 13 increased in frequency in the cerebellum upon conditional Chd4 knockout in a manner that correlated with increased genomic accessibility, H3K27 acetylation, and cohesin binding among enhancers within this domain (Fig. 2a, Supplementary Fig. 3F). On a genome-wide level, changes in genomic accessibility in contact domains also correlated with changes in contact domain interaction frequency upon conditional Chd4 knockout (Fig. 2b, c). Likewise, changes of contact domain interaction frequency upon conditional Chd4 knockout correlated with alterations in H3K27 acetylation and cohesin binding within these domains (Fig. 2d, e). The effects of conditional Chd4 knockout on contact domain interaction frequency were similar in both loop and compartmental domains (Supplementary Fig. 3G–I) and in domains called using the independent domain-calling algorithm TADtree (Supplementary Fig. 3J)^38^. In control analyses, contact domain interaction frequency upon conditional Chd4 knockout poorly correlated with changes in H2A.Z in these domains (Supplementary Fig. 3K), which is predominantly altered at promoters upon conditional Chd4 knockout^18^. Additionally, the changes in contact domain interaction frequency were independent of changes in boundary insulation (Supplementary Fig. 3L). Taken together, our data suggest that Chd4 regulates genomic interactions within contact domains in a manner that correlates tightly with Chd4 control of genomic accessibility and cohesin binding at enhancer sites in the brain.

Genome-wide interactions segregate into higher order contact patterns that are thought to reflect broad compartmentalization of the genome within the nucleus^21,25^. Because Chd4 coordinately regulates the epigenetic and interaction states of contact domains in the cerebellum, we next considered a role for Chd4 in contact domain compartmentalization in the brain. We first segregated each chromosome into 150-kb bins and assigned regions of the chromosome into active (A) and inactive (B) compartments based on the regional similarity in contact patterns across genomic loci (Supplementary Figs. 2D, 3M). We next assessed the relationship between changes in interaction frequency among domains and their corresponding compartmentalization. Upon visualizing compartmentalization of contact domains across the genome, we found concordant changes in contact domain interaction frequency and domain compartmentalization. An example contact domain on chromosome 13 was identified in a genome-wide bin with an eigenvalue of near zero (Fig. 2a), indicating weak partition of the contact domain into the A or B compartment. In the cerebellum of conditional Chd4 knockout mice, the eigenvalue of the compartmental bin was robustly increased (Fig. 2a), representing a shift of the contact domain into the A compartment. Consistent with this observation, using Pearson’s correlation matrix, the chromosome-wide interactions of the chromosome 13 region were more correlated with chromosome-wide interactions among A compartment regions than those in B compartment regions in the conditional Chd4 knockout cerebellum (Fig. 2a). Analysis of contact domain compartmentalization on a genome-wide level supported these observations. Compartmental bins demonstrated minimal genome-wide changes upon conditional Chd4 knockout (Supplementary Fig. 3N). Surprisingly, changes in contact domain interactions and epigenetic status strongly correlated with changes in compartmentalization of the domain (Fig. 2f). In other words, contact domains with increases in intra-domain interaction frequency, genomic accessibility, H3K27 acetylation, or cohesin occupancy became more associated with the A compartment upon conditional Chd4 knockout (Fig. 2f). These data show that Chd4 contributes to the compartmentalization of contact domains within the nucleus in the brain in accordance with the epigenetic state and domain interactions.

### Chd4 regulates loop domain boundary loop strength

**Fig. 3:**
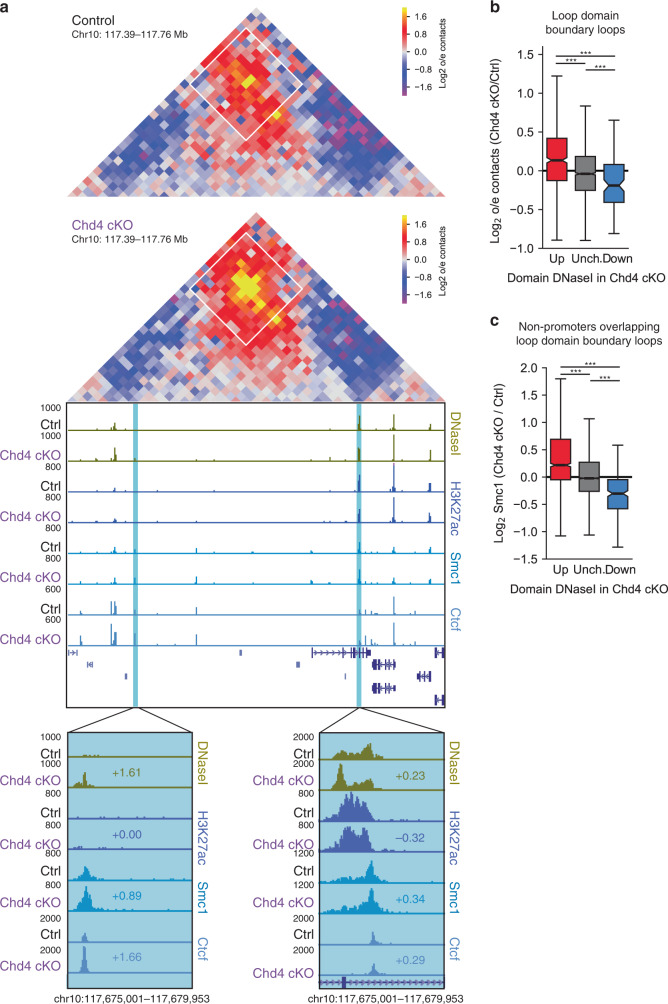
Chd4 regulates loop domain boundary loop strength. **a** (Top) Hi-C contact matrix of a loop domain on chromosome 10 and the flanking region, with a loop domain boundary loop highlighted by a white box. (Bottom) Genome-browser snapshot of the region corresponding to the Hi-C contact matrix displaying the ChIP-seq profiles of H3K27ac, Smc1, and Ctcf as well as DNaseI-seq from the control and Chd4 cKO cerebellum. Blue denotes the the loop anchors and regions of the insets. Numbers indicate the Log2 change in signal in the Chd4 cKO cerebellum. **b** Change in Hi-C contacts at loop domain boundary loops among domains with increased (*n* = 613), unchanged (*n* = 2299), or decreased (*n* = 105) accessibility in the Chd4 cKO. **c** Change in Smc1 occupancy at non-promoter DHS underlying loop domain boundary loops among domains with increased (*n* = 1343), unchanged (*n* = 5336), or decreased (*n* = 241) accessibility. *P*-values for all comparisons in this figure were calculated by the two-sided Kruskal–Wallis H-test for independent samples with Dunn’s post hoc T-test and corrected for multiple comparisons by the Bonferroni–Hochberg procedure. ***p < *0.001*.

Because Chd4 regulates binding of the cohesin complex, which is critical for loop formation^22,23^, we next asked whether Chd4 controls genomic looping events in the brain. Control of loop domain boundary loops is thought to contribute to the ability of looping to regulate contact domain interactions^22–24^. We found that conditional Chd4 knockout increased the interaction frequency of loop domain boundary loops in a manner that correlated with changes in epigenetic features at regulatory sites at loop domain boundaries in the mouse cerebellum. For example, a loop domain on chromosome 10 with increased domain accessibility upon conditional Chd4 knockout demonstrated increased interaction frequency at the loop domain boundary loop (Fig. 3a, Supplementary Fig. 4A). The increase in accessibility upon conditional Chd4 knockout in this loop domain occurred selectively at domain boundaries underlying the loop domain boundary loop (Fig. 3a). Accordingly, cohesin binding also increased at both loop anchors (Fig. 3a). Surprisingly, H3K27 acetylation minimally changed at the loop anchors (Fig. 3a), suggesting that distinct mechanisms might be involved in coordinating H3K27 acetylation and cohesin binding at enhancers. In other analyses, Ctcf occupancy increased at the upstream but not downstream loop anchor at the Chromosome 10 domain upon Chd4 depletion (Fig. 3a). Quantitative analysis of loop domain boundary loops genome-wide corroborated results observed at the chromosome 10 loop. The change in accessibility of a contact domain correlated with that of interaction frequency at loop domain boundary loops in the cerebellum of conditional Chd4 knockout mice (Fig. 3b, Supplementary Fig. 4B). Additionally, the change in accessibility of a contact domain correlated with that of cohesin binding upon conditional Chd4 knockout at the regions underlying loop domain boundary loops (Fig. 3c, Supplementary Fig. 4C). Notably, accessibility of contact domains correlated poorly with Ctcf binding upon conditional Chd4 knockout at the regions underlying loop domain boundary loops (Supplementary Fig. 4D). Taken together, these data suggest that Chd4 may regulate loop domain boundary loop interactions with alterations of cohesin binding at loop domain boundary loops.

### Chd4 coordinates intra-domain loops and gene expression

**Fig. 4:**
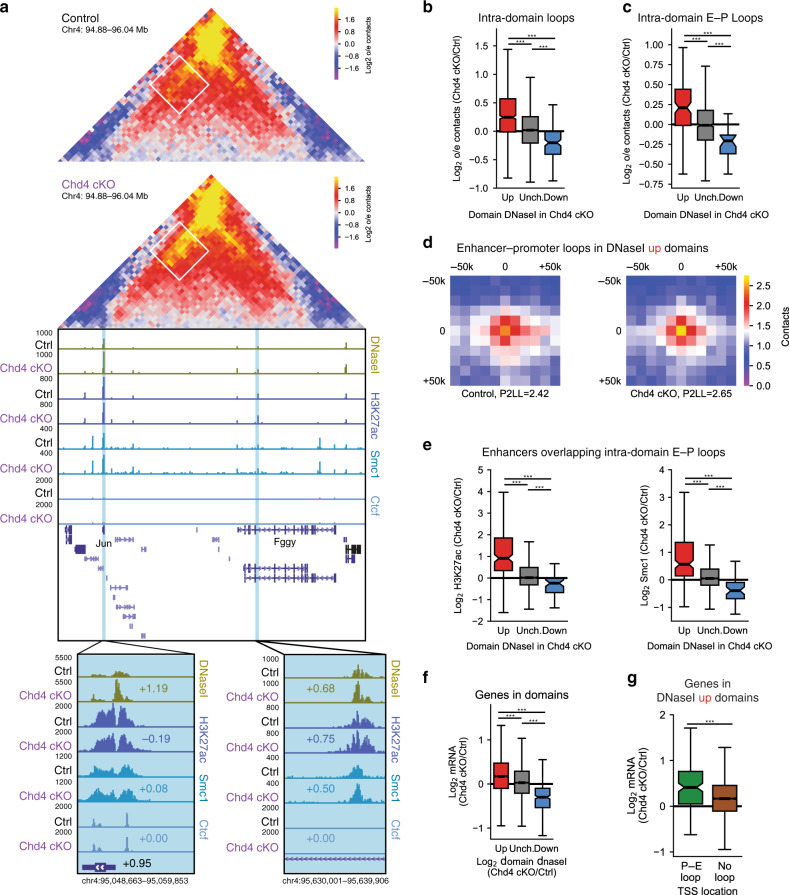
Chd4 coordinates intra-domain loop strength and gene expression. **a** (Top) Hi-C contact matrix of a loop domain on chromosome 4 and the flanking region, with an intra-domain enhancer-promoter (E–P) loop highlighted by a white box. (Bottom) Genome-browser snapshot of the region corresponding to the Hi-C contact matrix displaying the ChIP-seq profiles of H3K27ac, Smc1, and Ctcf as well as DNaseI-seq from the control and Chd4 cKO cerebellum. Blue denotes the the loop anchors and regions of the insets. Numbers indicate the Log2 change in signal in the Chd4 cKO cerebellum, including that of mRNA for *Jun*. **b**, **c** Hi-C contacts at intra-domain (**b**) or intra-domain E–P (**c**) loops among domains with increased (*n* = 938 intra-domain; *n* = 116 intra-domain E–P), unchanged (*n* = 4928 intra-domain; *n* = 953 intra-domain E–P), or decreased (*n* = 133 intra-domain; *n* = 26 intra-domain E–P) accessibility in the Chd4 cKO. Two-sided Kruskal–Wallis H-test for independent samples with Dunn’s post hoc T-test and corrected for multiple comparisons by the Bonferroni–Hochberg procedure. ***p < *0.001*. **d** Aggregate peak analysis of enhancer-promoter loops in domains with increased accessibility in the Chd4 cKO cerebellum. P2LL, peak-to-lower-left. **e** Change in (left) H3K27ac and (right) Smc1 at enhancers underlying intra-domain E–P loops among domains with increased (*n* = 183), unchanged (*n* = 1682), or decreased (*n* = 45) accessibility. Two-sided Kruskal–Wallis H-test for independent samples with Dunn’s post hoc T-test and corrected for multiple comparisons by the Bonferroni–Hochberg procedure. ***p < *0.001*. **f** Change in mRNA of genes in domains with increased (*n* = 3,202), unchanged (*n* = 12,649), or decreased (*n* = 311) accessibility in the Chd4 cKO. Two-sided Kruskal–Wallis H-test for independent samples with Dunn’s post hoc T-test and corrected for multiple comparisons by the Bonferroni–Hochberg procedure. ***p < *0.001*. **g** Change in mRNA of genes at intra-domain E–P loops (green, *n* = 145) or underlying no detectable loop (brown, *n* = 3057) in domains with increased accessibility in the Chd4 cKO. Two-sided Mann–Whitney U. ***p < *0.001*.

Besides loop domain boundary loops, we next asked if Chd4 might regulate other loop types in the cerebellum. Using the algorithm HiCCUPSDiff^39^, we identified 80 loops unique to the cerebellum of control mice and 203 loops unique to the cerebellum of conditional Chd4 knockout mice (Supplementary Fig. 5A, B). Surprisingly, the vast majority of loops distinct between control and conditional Chd4 knockout mice were intra-domain loops rather than loop domain boundary loops (Supplementary Fig. 5C). Further, among domains with increased accessibility, the interactions between intra-domain accessibility sites increased more than those at loop domain boundary loops upon conditional Chd4 knockout (Supplementary Fig. 5D). Analyses of intra-domain genomic loops revealed that conditional Chd4 knockout increased intra-domain loop interaction frequency in the cerebellum in a manner that correlated with changes of epigenetic features of regulatory sites within the domain. For example, conditional Chd4 knockout increased the interaction frequency of an intra-domain enhancer-promoter loop in a chromosome 4 contact domain in the cerebellum (Fig. 4a, Supplementary Fig. 5E). The loop connected a region containing the promoter of the *Jun* gene to a set of enhancers in an intron of the *Fggy* gene (Fig. 4a). Notably, Ctcf was present at the *Jun* promoter but not at intronic enhancers in the *Fggy* gene (Fig. 4a). Conditional Chd4 knockout increased accessibility at the *Jun* promoter with minimal changes in H3K27 acetylation or binding of cohesin and Ctcf (Fig. 4a). In contrast, conditional Chd4 knockout increased both accessibility and cohesin binding at the intronic enhancers in the Fggy gene in the cerebellum (Fig. 4a). Similar epigenetic and looping changes occured at an intra-domain enhancer-promoter loop connecting an enhancer within the *Gm13807* locus to the promoter of the *Tspan18* gene (Supplementary Fig. 5F). Genome-wide analysis of intra-domain loops revealed that changes in interaction frequency at intra-domain loops correlated with changes in accessibility within the domain upon Chd4 depletion (Fig. 4b). Additionally, intra-domain loops unique to the cerebellum of conditional Chd4 knockout mice were enriched among domains with increased accessibility (Supplementary Fig. 5C). These analyses suggest that Chd4 may preferentially coordinate intra-domain loops genome-wide with alterations in domain accessibility.

To further assess the mechanism through which Chd4 might regulate loop formation within contact domains, we assessed the relationship between epigenetic changes and looping specifically at intra-domain enhancer-promoter loops. Similar to all intra-domain loops, intra-domain enhancer-promoter loop interaction frequency correlated with changes in accessibility within a contact domain upon Chd4 loss (Fig. 4c). Intra-domain enhancer-promoter loops within contact domains with increased accessibility displayed increased interaction frequency in the cerebellum of conditional Chd4 knockout mice (Fig. 4d). Likewise, changes in contact domain accessibility correlated more strongly with changes in H3K27 acetylation and cohesin binding at enhancers than at promoters underlying enhancer-promoter loops in the cerebellum of conditional Chd4 knockout mice (Fig. 4e, Supplementary Fig. 5G–J). Together, these data show that Chd4 may regulate intra-domain enhancer-promoter loop interaction frequency via changes in the epigenetic state and cohesin binding of enhancers underlying these loops.

The role of Chd4 in intra-domain enhancer-promoter loops led us to ask whether Chd4 regulates expression of genes located within contact domains. Analysis of gene expression changes in the cerebellum in conditional Chd4 knockout mice revealed that increases in accessibility in a contact domain correlated with increased expression of genes localized within the domain (Fig. 4f)^16^. In addition, genes harboring intra-domain enhancer-promoter loops in domains with increased accessibility were more robustly upregulated in the cerebellum upon Chd4 depletion than genes without detectable promoter-enhancer loops at the 6 kb Hi-C resolution (Fig. 4g). Expression of genes with intra-domain enhancer-promoter loops located in domains with unaltered or decreased accessibility upon Chd4 loss changed similarly to those without detectable loops (Supplementary Fig. 5K, L). Together, these data suggest that Chd4 represses gene expression by reducing the strength of intra-domain enhancer-promoter loops in contact domains.

### Chd4 controls the epigenomic maturation of contact domains

**Fig. 5:**
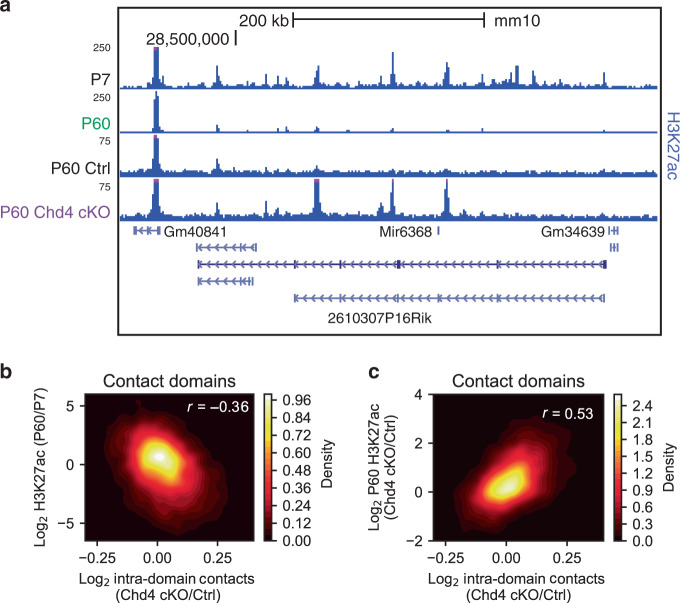
Chd4 loss impairs the maturation of epigenomic features in contact domains. **a** Genome-browser snapshot of a contact domain on chromosome 13 as in Fig. 2a displaying the ChIP-seq profiles of H3K27ac in the P7 and P60 cerebellum. Also displayed is the ChIP-seq profile of H3K27ac in the cerebellum of control and Chd4 cKO mice at P60. Blue denotes the extent of the contact domain. **b**, **c** Density plot comparing the change in Hi-C contacts within a domain with the (**b**) change in H3K27ac in the cerebellum between P7 and P60 and (**c**) change in H3K27ac in the cerebellum of control and Chd4 cKO mice at P60. Pearson’s *r*, *p* < 0.001.

Chd4 plays a critical role in the developmental regulation of gene expression in neurons and consequent establishment of neuronal connectivity^16,18^. We therefore assessed whether Chd4 controls neuronal genome architecture in developmentally regulated contact domains in the brain. We found that contact domains with altered interactions in the cerebellum upon conditional Chd4 knockout contained regulatory sites that were dynamic during brain development. Upon visualization of contact domains with increased accessibility and interaction frequency in the cerebellum of conditional Chd4 knockout mice, H3K27 acetylation at enhancers across the contact domain in the cerebellum of wild-type mice diminished robustly from P7 to P60 (e.g. chromosome 13, Fig. 5a)^40^. Genome-wide analysis revealed that the change in interaction frequency of contact domains following conditional Chd4 knockout was correlated with downregulation of H3K27 acetylation in the cerebellum from P7 to P60 (Fig. 5b, Supplementary Fig. 6A). These data led us to consider a role for Chd4 in the maturation of epigenetic features in contact domains in the cerebellum. To determine whether Chd4 controls the timing or maturation of epigenetic features in contact domains in the brain, we performed ChIP-seq analyses of H3K27 acetylation at P60 in the cerebellum of control and conditional Chd4 knockout mice (Fig. 5a). Remarkably, contact domains with increased accessibility and interaction frequency demonstrated increased H3K27 acetylation at P60 in the cerebellum of conditional Chd4 knockout mice (Fig. 5c, Supplementary Fig. 6B, C). Additionally, the change in H3K27 acetylation at P22 remained similarly changed at P60 in the cerebellum of conditional Chd4 knockout mice (Supplementary Fig. 6D). These results suggest that Chd4 may gate the maturation of epigenetic features in contact domains in the cerebellum.

We next considered a role for Chd4 in the maturation of genomic compartments in the cerebellum. We found that contact domains that became more strongly associated with the A compartment upon conditional Chd4 knockout contained regulatory sites that were developmentally inactivated (Supplementary Fig. 6E). Conversely, contact domains that became more strongly associated with the B compartment upon conditional Chd4 knockout contained regulatory sites that were developmentally activated (Supplementary Fig. 6E). Together, these data suggest that Chd4 controls the maturation of the epigenetic and architectural status of the genome in the brain.

## Discussion

**Fig. 6:**
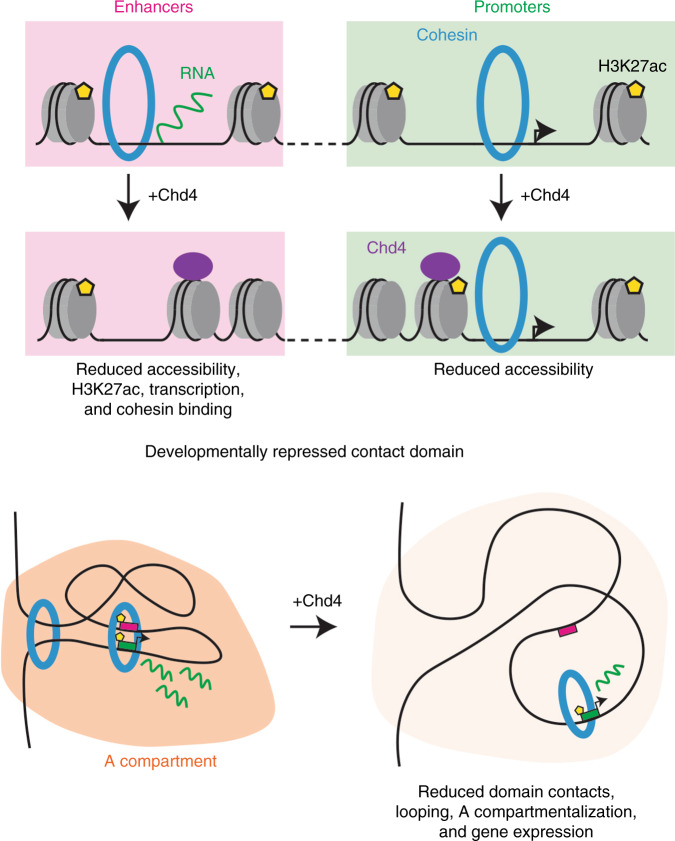
Chd4 in the control of enhancer function and genome architecture. (Top) Conditional knockout of Chd4 (purple) in granule neurons of the mouse cerebellum increases the accessibility of gene enhancers (pink) and promoters (mint) genome-wide in vivo. Conditional Chd4 knockout promotes acetylation of histone H3K27 (yellow pentagons), transcription of enhancer RNAs (green lines), and cohesin complex (blue) binding specifically at gene enhancers. (Bottom) Profiling of genome architecture in vivo shows that conditional knockout of Chd4 strengthens domain contacts, looping at loop domain boundaries and between promoters (green) and enhancers (pink), A compartmentalization (orange), and gene expression (green lines) among developmentally repressed contact domains.

In this study, we have discovered a role for Chd4 in the organization of genome architecture in the mammalian brain (see model in Fig. 6). Conditional knockout of Chd4 in granule neurons of the mouse cerebellum increases the accessibility of gene enhancers and promoters genome-wide in vivo. Remarkably, conditional Chd4 knockout promotes recruitment of the architectural protein cohesin selectively to gene enhancers in granule neurons in vivo. Importantly, profiling of genome architecture in vivo demonstrates that conditional knockout of Chd4 strengthens interactions among developmentally repressed contact domains and genomic loops in a manner that correlates with changes in the epigenetic and gene expression status of regions underlying these architectural features. In sum, our findings define a role for chromatin remodeling in the organization of genome architecture in the brain in vivo.

Our findings have broad implications in our understanding of the role of chromatin remodeling enzymes in the control of the epigenome and genome architecture. In this study, we have discovered roles for Chd4 in the control of gene enhancers in the brain in vivo. A fundamental question that remains to be addressed in genome biology is how chromatin remodeling enzymes might impact the distinct actions of promoters and enhancers in the control of gene expression. We have found that Chd4 reduces accessibility genome-wide at both enhancers and promoters in the brain in vivo. However, changes in accessibility at promoters and enhancers may trigger distinct epigenomic consequences at these sites. Chd4 decommissions the promoters of a select set of developmentally regulated genes by regulating histone tail modification^16^; and Chd4 stimulates deposition of the histone variant H2A.z at promoters of neuronal activity genes and thereby triggers their dynamic acute shutoff^18^. By contrast, here we have uncovered that Chd4 strikingly represses enhancers and inhibits recruitment of the genome architectural protein complex cohesin at a genome-wide level in the brain in vivo. In future studies, it will be important to determine the mechanisms by which Chd4 differentially regulates gene enhancers and promoters.

Our study also reveals that chromatin remodeling may influence genome architecture in the brain. Chd4 suppresses interactions within contact domains in the developing brain, such that these domains shift compartmentalization in the nucleus upon conditional Chd4 knockout. Chd4 may conceivably weaken genomic interactions within these domains via restriction of cohesin binding at enhancers within contact domains. In future studies, it will be important to determine how Chd4 inhibits cohesin binding at gene enhancers. Because the cohesin loading complex Nipbl binds to active enhancers in the developing cortex^41^, it will be interesting to determine whether Chd4 regulates Nipbl function at enhancers. Alternatively, Chd4 might alter the binding of a transcription factor that in turn controls cohesin occupancy at gene enhancers^32^. It will be additionally important to determine whether Chd4 controls looping strength directly through altered enhancer activity or as a consequence of enhancer-driven changes in gene expression. Directed looping of enhancers to gene promoters can upregulate gene expression^42,43^. By contrast, enhancer activity might coordinate promoter activity independently of genomic looping^44^.

The control of genome architecture may be fundamental to the development of the brain. Widespread changes in contact domain, loop, and compartmental interactions accompany neuronal differentiation in vivo^26^, suggesting that the activity of regulatory sites may be critical to the developmental maturation of genome architecture. Conditional knockout of Chd4 in the brain in vivo increases interactions within developmentally repressed contact domains, reflected further in the shift in compartmentalization of these domains into the active compartment. The control of genome architecture by Chd4 may thus play a crucial role in maturation of the epigenome of neurons in the brain. These findings are particularly relevant to our understanding of neurodevelopmental disorders of cognition. Mutations of Chd4 and other chromatin remodeling enzymes as well as of proteins closely associated with genome architecture including the cohesin complex cause neurodevelopmental disorders of cognition such as intellectual disability and autism^10–12,27,45–54^. Interestingly, truncating mutations in the cohesin loading complex protein Nipbl cause severe clinical features^27^, suggesting that failure to load cohesin may be critical to brain development. Conversely, conditional Chd4 knockout leads to an increase in cohesin binding to enhancers en masse, suggesting that balanced level of cohesin occupancy on the genome and hence level of genomic interactions may be critical to brain development. Dysregulation of genome architecture may thus constitute a key mechanism by which mutations in chromatin regulators lead to neurodevelopmental disorders of cognition including autism spectrum disorders and intellectual disability.

## Methods

### Animal husbandry

Control (Chd4^f/f^) and Chd4 cKO (Chd4^f/f^; GABRA6-Cre^+/−^) mice^16,18,55^ were housed under pathogen-free conditions. Experiments were performed in accordance with protocols approved by the Animal Studies Committee at Washington University in St. Louis School of Medicine and National Institutes of Health guidelines.

### Antibodies

Smc1 5 µg/100 µL lysate (Bethyl A300-055A), Ctcf 3 µg/200 µL lysate (Millipore 07-729), H3K27ac 0.1 µL/500 µL lysate (Abcam ab4729), and H3K4me1 3 µL/500 µL (Active Motif 39297) antibodies were used in this study.

### DNaseI-seq

DNaseI-seq was performed as previously described^56^. The cerebellum was dissected and homogenized in dissection buffer (20 mM MOPS, 40 mM NaCl, 90 mM KCl, 2 mM EDTA, 0.5 mM EGTA, 0.5 mM Spermidine, 0.2 mM Spermine) then passed through a 70um filter. Tissue lysates were then mixed into 2 M sucrose (final 1.74 M) and centrifuged at 23krpm in an SW40T rotor (Beckman Coulter) at 4 °C for 1 h. Nuclear pellets were then resuspended in digestion buffer (750 mM NaCl, 60 mM CaCl_2_) to a concentration of 10 million nuclei/mL. Five million nuclei were pre-warmed at 37 °C for 1 min then treated with DNaseI (20U) at 37 °C for 3 min. The reaction was stopped by addition of stop buffer (final 25 mM Tris-HCl pH 7.5, 50 mM NaCl, 0.05% SDS, 50 mM EDTA, 0.5 mM spermidine, 0.15 mM spermine, proteinase K [NEB]) then incubated at 55 °C for 1 h. The reaction was then treated with RnaseA at 37 °C for 30 min. Samples were gently mixed with phenol-chloroform then centrifuged to obtain the supernatant. The supernatant was mixed with NaCl (final 798 mM) and fractionated using a sucrose cushion (10, 20, 30, 40% [w/v]) by centrifugation at 25krpm in an SW40T rotor at 4 °C for 24 h. Fractions with less than 500 bp DNA fragments were purified using a PCR purification kit (Qiagen) and sequenced on an Illumina HiSeq 2000 (Genome Technology Access Center at Washington University). Two biological replicates of sex-matched littermates per condition were used for DNaseI-seq experiments.

### ChIP-seq

ChIP-seq was performed as previously described with minor modifications^18^. For Ctcf ChIP-seq, the cerebellum was dissected and homogenized in a 1.01% formaldehyde solution (4.5 mM HEPES-KOH pH 7.9, 9.1 mM NaCl, 0.09 mM EDTA, 0.05 mM EGTA, 0.9X PBS) while rotating for 15 min at room temperature (RT). The formaldehyde was quenched with the addition of Tris and glycine (final 113 mM glycine, 0.91 mM Tris-HCl) while rotating for 5 min at RT. The cell pellet was washed with cold 1X PBS then flash frozen and stored at −80 °C.

For Smc1 ChIP-seq, the cerebellum was dissected and homogenized in a 2 mM disuccinimidyl glutarate (DSG; ThermoFisher) dissolved in 1X PBS while rotation for 45 min at RT. Tissue was pelleted then washed twice with 1X PBS at RT. Tissue was then resuspended in a 1.01% formaldehyde solution (4.5 mM HEPES-KOH pH 7.9, 9.1 mM NaCl, 0.09 mM EDTA, 0.05 mM EGTA, 0.9X PBS) while rotating for 15 min at room temperature (RT). The formaldehyde was quenched with the addition of Tris and glycine (final 113 mM glycine, 0.91 mM Tris-HCl) while rotating for 5 min at RT. The cell pellet was washed with cold 1X PBS then flash frozen and stored at −80 °C.

For H3K4me1 and P60 H3K27ac ChIP-seq, the cerebellum was dissected and homogenized in a 1.01% formaldehyde solution in 1X PBS for 14 min. Formaldehyde was quenched with glycine (130 mM) for 5 min at RT. The cell pellet was resuspended in cold NP-40 buffer (0.1% NP-40 in 1X PBS) then filtered through a 40 μm cell strainer. Cell pellets were then washed twice with cold NP-40 buffer then flash-frozen and stored at −80 °C.

Immunoprecipitation was performed in RIPA buffer (10 mM Tris-HCl 8.0, 140 mM NaCl, 0.1% SDS, 1% Triton-X100, 0.1% DOC, 1 mM EDTA, 0.5% EGTA) with the respective antibody and beads. For Smc1, Dynabeads protein G (ThermoFisher Scientific) were used; for Ctcf, Sepharose protein G beads (GE Life Sciences) were used; for H3K27ac, Dynabeads protein G and protein A (ThermoFisher Scientific) beads were used; for H3K4me1, Sepharose protein G and protein A (GE Life Sciences) were used. Smc1, H3K27ac, and H3K4me1 ChIP-seq libraries were prepared using the Swift NGS 2S Plus Library Prep Kit per kit instructions. Ctcf ChIP-seq libraries were prepared using the NEBNext Ultra II DNA Library Prep Kit for Illumina (NEB) per kit instructions. Smc1 libraries were sequenced on an Illumina HiSeq 2500; Ctcf, H3K27ac, and H3K4me1 libraries were sequenced on an Illumina NextSeq 500. Two biological replicates of sex-matched littermates per condition (Smc1: 2F; H3K4me1: 1F, 1M; P60 H3K27ac: 2F) were used for ChIP-seq experiments.

### Nuclear RNA-seq

The cerebellum was dissected, washed with cold 1X PBS, then homogenized in cold lysis buffer (25 mM HEPES pH 7.4, 140 mM NaCl, 0.1% NP-40, 1.5 mM MgCl_2_) with 200 U/mL RNase Inhibitor (New England Biolabs). Nuclei were then filtered with a 40 μm cell strainer then pelleted by gentle centrifugation. Nuclear pellets were washed with cold lysis buffer then resuspended in 1 mL Trizol (Invitrogen). RNA was then purified per Trizol reagent instructions. rRNA was depleted from RNA samples using the NEBNext rRNA Depletion Kit (NEB) per kit instructions. RNA-seq libraries were generated using NEBNext Ultra Directional RNA Library Prep Kit for Illumina (NEB) per kit instructions. DNA was sequenced on the NextSeq 500 (Center for Genome Sciences at Washington University). Four biological replicates of sex-matched littermates per condition (2 F, 2 M) were used for nuclear RNA-seq experiments.

### Hi-C

Hi-C was performed as previously described with minor modifications^56^. The cerebellum was dissected and homogenized in a 1.01% formaldehyde solution (4.5 mM HEPES-KOH pH 7.9, 9.1 mM NaCl, 0.09 mM EDTA, 0.05 mM EGTA, 0.9X PBS) while rotating for 15 min at room temperature (RT). The formaldehyde was quenched with the addition of Tris and glycine (final 113 mM glycine, 0.91 mM Tris-HCl) while rotating for 5 min at RT. The cell pellet was washed with cold 1X PBS then flash frozen and stored at −80 °C.

Flash-frozen tissue pellets were thawed on ice then resuspended in 15 mL cold lysis buffer (10 mM Tris-HCl pH 8, 10 mM NaCl, 0.2% IGEPAL-630 with proteinase inhibitors) and incubated on ice for 15 min. Tissue was then homogenized, passed through a 70 μm nylon filter, pelleted, then washed with lysis buffer to purify nuclei. Nuclei were then resuspended in 2.5 mL 0.5% SDS and incubated at 62 °C for 10 min to permeabilize nuclei. 100-250k nuclei from this suspension (25 μL) of this nuclear suspension was then quenched with a Triton-X100 solution (final 1% Triton-X100, 1.2% Cutsmart buffer [NEB]) and incubated at 37 °C for 15 min. Nuclei were then treated with MboI (50U; NEB) and spun at 300 rpm at 37 °C for 4 h, followed by incubation at 65 °C for 20 min to inactivate the enzyme.

DNA blunting was performed by incubating nuclei with Biotin-14-dATP and other dNTPs (final 30 μM) with Klenow (20U; NEB) at 300 rpm at 37 °C for 4 h. Proximity ligation was performed by incubating nuclei in a ligation buffer (final 1X T4 DNA ligase buffer [NEB], 0.1 mg/mL Bovine Serum Albumin [BSA], 1% Triton-X100) with T4 DNA Ligase (4000U) at 300 rpm at 16 °C overnight. Nuclei were then pelleted and resuspended in 1X Cutsmart buffer (NEB). SDS (final 0.8%), NaCl (final 217 mM), and proteinase K (3.2U; NEB) were then added and spun at 1200 rpm at 55 °C for 1 h, then at 1200 rpm at 65 °C for >12 h. RnaseA (0.02 mg; ThermoFisher Scientific) was then added and incubated at 37 °C for 1 h. DNA was purified by phenol-chloroform purification followed by ethanol precipitation in the presence of glycogen. Biotin was removed from free ends in a dATP solution (100 μM dATP, 1X Buffer 2.1 [NEB]) with 1 U/μg DNA T4 DNA Polymerase (NEB) at 20 °C for 4 h. DNA was then purified using a Monarch PCR & DNA Cleanup Kit (NEB).

Purified DNA was sonicated to 300 bp using a Covaris E220 instrument. Sonication tubes were washed with an additional volume of TE to capture DNA stuck to side of tubes. Right-sided size selection was performed using SPRIselect beads.

Biotin-labeled DNA was captured using Dynabeads MyONE Streptavidin T1 (ThermoFisher). Beads were then resuspended in 40 μL Low-EDTA TE (Swift Biosciences) and used in the Swift NGS 2 S Plus Library Prep Kit (Swift Biosciences) with minor modifications. For all washes, beads were resuspended in 2X TBW (10 mM Tris-HCl pH 8, 1 mM EDTA, 2 M NaCl, 0.05% Tween-20), incubated for 5 min at RT, then washed twice with 1X TBW. Beads were then resuspended in the appropriate volume of enzyme master mix (Swift Biosciences) for each step. Prior to amplification, DNA was eluted from beads by incubation in Low-EDTA TE at 98 °C for 10 min. DNA was then amplified using 14 cycles of PCR according to kit instructions. Following amplification, cDNA was sequenced on the NextSeq 500 (Center for Genome Sciences at Washington University). Three biological replicates of sex-matched littermates per condition (3 M) were used for Hi-C experiments.

### Data analysis

DNaseI-seq and ChIP-seq reads were aligned to mm10 using Bowtie2 (v2.2.5)^57^. Conversion of sam to bam files was performed using Samtools (v1.3)^58^. DNaseI-seq reads per kilobase per million (rpkm) was quantified using Deeptools (v2.4.2) bamCoverage with no extension^59^. ChIP-seq rpkm was quantified using Deeptools bamCompare assuming a 300 bp fragment size (−e 300) with reads centered (--centerReads) and input subtracted. For P22 formaldehyde-treated ChIP-seq samples, inputs^16,18^ were concatenated and used as a single input. Signal was then divided by the binsize to generate DNaseI- or ChIP-seq reads per million (rpm). To generate genomic tracks for viewing on the UCSC genome browser, DNaseI-seq biological replicates were concatenated using Samtools then quantified as described above^60^. ChIP-seq reads were concatenated using Samtools, then rpkm was quantified using Deeptools bamCoverage assuming a 300 bp fragment size (−e 300) with reads centered (--centerReads). Tracks are represented using a two-pixel smoothing window. Individual biological replicates were compared using Deeptools multiBigwigSummary and plotCorrelation on Galaxy (v3.3.0.0.0) with 10 kb bins and the Spearman correlation method. Clustering was performed with Seaborn (v0.9.0) clustermap using default settings.

Nuclear RNA-seq sequences were adapter-trimmed using Cutadapt (v1.16)^61^ and subjected to quality control using PRINSEQ (v0.20.4)^62^ and aligned to mouse genome mm10 using STAR (v2.5.3a)^63^. Sequencing performance was assessed for total number of aligned reads, total number of uniquely aligned reads, genes and transcripts detected, ribosomal fraction, known junction saturation, and reads distribution over known gene models with Picard Tools (v2.19.0) (http://broadinstitute.github.io/picard/), qualimap (v2.2.1)^64^, RSeQC (v2.6.2)^65^.

HiC-Pro (v2.10.0) was used to generate contact matrices using the mm10 mouse genome as reference^66^. Valid pairs determined by HiC-Pro was used as input to generate Hi-C contact matrices at 1, 5, 10, 20, 40, 150, 500 kb and 1 million base pair resolutions. addNorm function from Juicer (v1.5.6) was used to perform genome-wide normalization^39^. Observed over expected (O/E) and Knight-Ruiz (KR)-normalized Hi-C contacts from genomic bins were extracted using juicer-tools (v1.8.9) dump. For visualization of contact matrices in Supplementary Fig. 3A, E, KR-normalized Hi-C contacts were extracted from genomic bins using juicer-tools (v1.8.9) straw then scaled to the same sequencing depth (Supplementary Fig. 3A, E); otherwise, KR-normalized, O/E Hi-C contacts were extracted from genomic bins using juicer-tools (v1.8.9) dump. Visualization of the Pearson’s matrix was performed using Juicebox (v1.9.8) at 40 kb resolution^39^.

The similarity of biological replicates was compared using three methods. Unnormalized contacts across all chromosomes at 1 Mb resolution were used to calculate the Spearman’s Rho. For HiCRep^67^ and HiC-Spector^68^ analyses, the quality score was calculated using 3DChromatinReplicate_QC (v1.01)^69^. The average correlation metric for HiCRep and HiC-Spector across all chromosomes was depicted in the clustered heatmap. Clustering was performed with Seaborn (v0.9.0) clustermap using default settings.

DNaseI-seq peaks were called using MACS2 (v2.1.1.20160309) at a q-value of less than 0.01 (−q 0.01) without model building (--nomodel), an extension of 200 bp (--extsize 200), and a shift of −100bp (--shift −100)^70^. Peaks from control and Chd4 cKO were then merged and called as significantly different using Diffbind (v2.6.6) running DESeq2 (v1.20.0)^71,72^. H3K4me1 ChIP-seq peaks were called using MACS2 (v2.1.1.20160309) using the broad settings (--broad) at a q-value of less than 0.05 (−q 0.05 –broad-cutoff 0.05) without model building (--nomodel) and an extension of 300 bp (--extsize 300). Active promoters were defined as transcription start sites overlapping DnaseI-seq and H3K27ac peaks. Active enhancers were defined as DnaseI-seq peaks overlapping H3K27ac and H3K4me1 peaks. Overlapping peaks were those that overlapped by at least 25% reciprocally (-f 0.25 -r) using Bedtools (v2.25.0)^73^. DNaseI- and ChIP-seq signal at DHS was quantified as the average signal among biological replicates in the 1 kb around the TSS or center of the DRE. Examples of these values are represented in the genome-browser snapshots.

RNA-seq reads in features were counted using HTseq (v0.6.1)^74^. Reads in exons were used to quantify gene abundance in mRNA-seq data. Enhancer RNAs (eRNAs) were identified using bidirectional windows originating from DnaseI-seq peak centers that overlapped with H3K27ac- and H3K4me1-marked enhancers and spanning 2 kb upstream on the Crick (−) strand or 2 kb downstream on the Watson (+) strand. Windows overlapping with known coding regions and lncRNAs (with 1 kb extension from both transcription start site and transcription end site) were excluded from analysis. DESeq2 (v1.26.0)^72^ was used to estimate the change in feature expression between conditions. Features with fewer than ten counts were removed from all analyses.

Genomic loops were identified from control and Chd4 cKO Hi-C data independently on the pooled set of KR-normalized contact matrices using juicer-tools (v1.9.9) HiCCUPS at 10 kb resolution on a CPU (--cpu) using default parameters^21^. Control and Chd4 cKO loops were then merged if both anchors were within 10 kb of one another. Significantly different genomic loops between control and Chd4 cKO Hi-C data were identified using juicer-tools HiCCUPSDiff on the pooled set of KR-normalized contact matrices at an FDR of less than 0.1 (−f 0.1) on a CPU (--cpu) using default parameters^39^. Loop contacts were quantified as the O/E, KR-normalized signal in the region defined by the loop anchor boundaries.

Contact domains were identified from control and Chd4 cKO Hi-C data independently on the pooled set of KR-normalized contact matrices using juicer-tools (v1.9.9) Arrowhead at 10 kb resolution using default parameters^21^. Control and Chd4 cKO domains were then merged if domain borders were within 20 kb of one another. Domains were identified as loop domains if domain boundaries were within 25 kb of loop anchors. Otherwise, they were termed ordinary domains. Similarly, loops were identified as domain loops if they were within 25 kb of domain boundaries. Otherwise, they were termed ordinary loops. Domain contacts were quantified as the O/E, KR-normalized signal in the region defined by the domain boundaries. DNaseI- and ChIP-seq signal within domains was quantified using the average signal of all DHS within the domain. Insulation scores for domain boundaries were calculated as the negative log-ratio of contacts that violate insulation compared to all contacts in the length of the domain on both sides of the boundary^75^. TADs in Supplementary Fig. 3J were identified using TADtree^38^ as previously described^76^ using KR-normalized contact matrices at 40 kb resolution with gamma = 200, *M* = 1, *p* = 3, *q* = 12, *N* = 500, and range = 1:500. TADs were only considered if present in at least 30% of all runs.

The eigenvalue was calculated from control and Chd4 cKO Hi-C data independently on the pooled set of KR-normalized contact matrices using juicer-tools (v1.9.9) eigenvector at 150 kb resolution on each individual chromosome. The sign of the eigenvector for each condition was then oriented so that positive value bins were correlated with H3K27ac signal in the bin. Contact domains were assigned the average eigenvalue of the 150 kb bins that the domain spanned.

To compare changes in interaction frequency between loop domain boundary loops and intra-domain DNaseI peaks found in domains with increased accessibility, DNaseI peaks within the same domain were paired. Distance-controlled resampling was then performed to generate one-hundred distributions of paired DNaseI peaks such that the pairs of DNaseI peaks selected matched the test set of loop domain boundary loop anchors in both number and distance between pairs. An empirical *p*-value of 0.01 was derived from the observation that one resampled distribution out of the one-hundred distributions analyzed showed a median fold-change in interactions less than the test distribution. The fourty-ninth of one-hundred resampled distributions sorted by the resampled distributions’ medians is presented in Supplementary Fig. 5D.

All analyses were performed with Jupyter notebook (v1.0.0) running Pandas (v0.24.2), Numpy (v1.16.2), and Scipy (v1.2.1) data analysis tools as well as Matplotlib (v2.2.4), Matplotlib-Venn (v0.11.5), and Seaborn (v0.9.0) plotting packages unless otherwise noted^77–80^. Among all boxplots, central lines represent the median, notches represent the 95% confidence interval of the median, boxes represent the 25th and 75th percentiles, and whiskers represent 1.5× the inter-quartile range.

### Reporting summary

Further information on research design is available in the Nature Research Reporting Summary linked to this article.

## Supplementary information

Supplementary Information

Reporting Summary

## Data Availability

The data generated in this study are available at GSE138822. All other relevant data supporting the key findings of this study are available within the article and its Supplementary Information files or from the corresponding author upon reasonable request. Published data used in this study are at the following DOIs: P7 and P60 H3K27ac ChIP-seq at GSE60731^40^; P22 Control and Chd4 cKO mRNA-seq and H3K27ac ChIP-seq at GSE57758^16^; and P22 Control and Chd4 cKO Chd4, H3K27me3, H2A.Z and H3 ChIP-seq at GSE83253^18^. Select source data, exact test statistics and p-values are provided in the source data file. A reporting summary for this Article is available as a Supplementary Information file. Source data are provided with this paper.

## Source data

Source Data

## Code availability

Code for generating figures are available upon request. Source data are provided with this paper.
