# Supplementary material for: The chromatin remodeling enzyme Chd4 regulates genome architecture in the mouse brain
Source: Nat Commun. 2020 Jul 9;11:3419. doi: 10.1038/s41467-020-17065-z (PMC7347877; doi:10.1038/s41467-020-17065-z)
Supplement: Supplementary file 1 — Supplementary Information [file 41467_2020_17065_MOESM1_ESM.pdf]

Supplementary Information

The Chromatin Remodeling Enzyme Chd4 Regulates Genome Architecture in the Mouse Brain

Goodman *et al.*

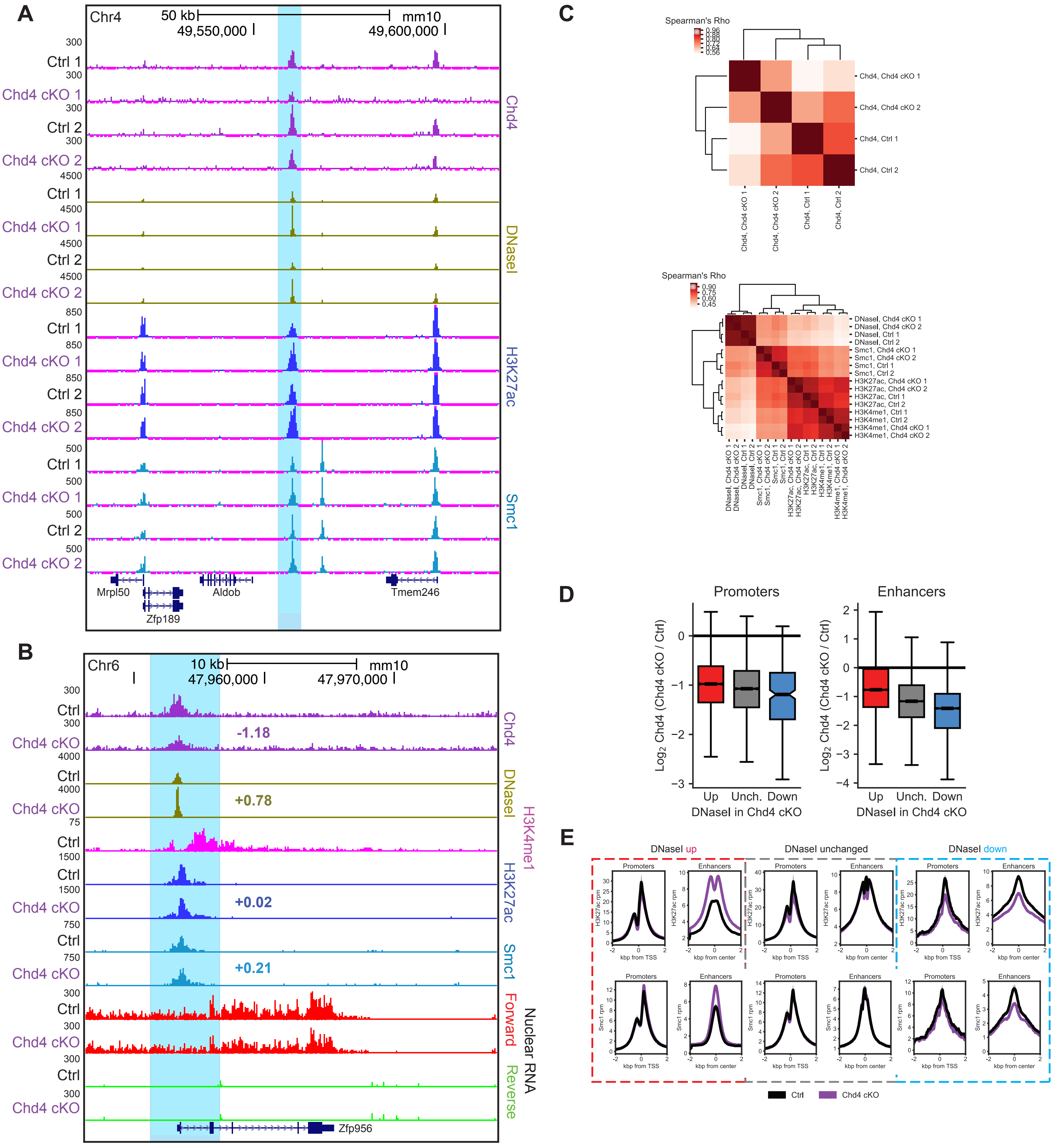

Supp Figure 1

Supplementary Figure 1: Chd4 depletion preferentially modulates enhancer activation and Cohesin binding at enhancers

(A) Genome browser snapshot of a region containing the *Aldob* gene locus on chromosome 4 displaying the ChIP-seq profiles of Chd4, H3K4me1, H3K27ac and Smc1 as well as DNaseI-seq from the control and Chd4 cKO cerebellum in biological replicates. Light blue denotes an enhancer upstream of the *Aldob* gene. **(B)** Genome browser snapshot of the promoter of the *Zfp256* gene locus on chromosome 6 displaying the ChIP-seq profile of Chd4, H3K4me1, H3K27ac, and Smc1 as well as DNaseI-seq and nuclear RNA-seq from the control and Chd4 cKO cerebellum. Numbers indicate the Log2 change in signal in the Chd4 cKO cerebellum. **(C)** Clustering of biological replicates from the control and Chd4 cKO cerebellum using Spearman's Rho from Chd4 (top) as well as DNaseI, H3K27ac, H3K4me1, and Smc1 (bottom). **(D)** Change in Chd4 between Chd4 cKO and control cerebellum at promoters and/or enhancers with increased (n=19,389 promoters; n=67,245 enhancers), unchanged (n=15,141 promoters; n=44,661 enhancers), and decreased (n=828 promoters, n=27,750 enhancers) accessibility. **(E)** Aggregate plot of (top) H3K27ac and (bottom) Smc1 read density in the control and Chd4 cKO cerebellum at promoters and enhancers with increased (left), unchanged (middle), and decreased (right) accessibility. P-values for all comparisons in this figure were calculated by the two-sided Kruskal-Wallis H-test for independent samples with Dunn's post hoc T-test and corrected for multiple comparisons by the Bonferroni-Hochberg procedure. ***$p<0.001$.

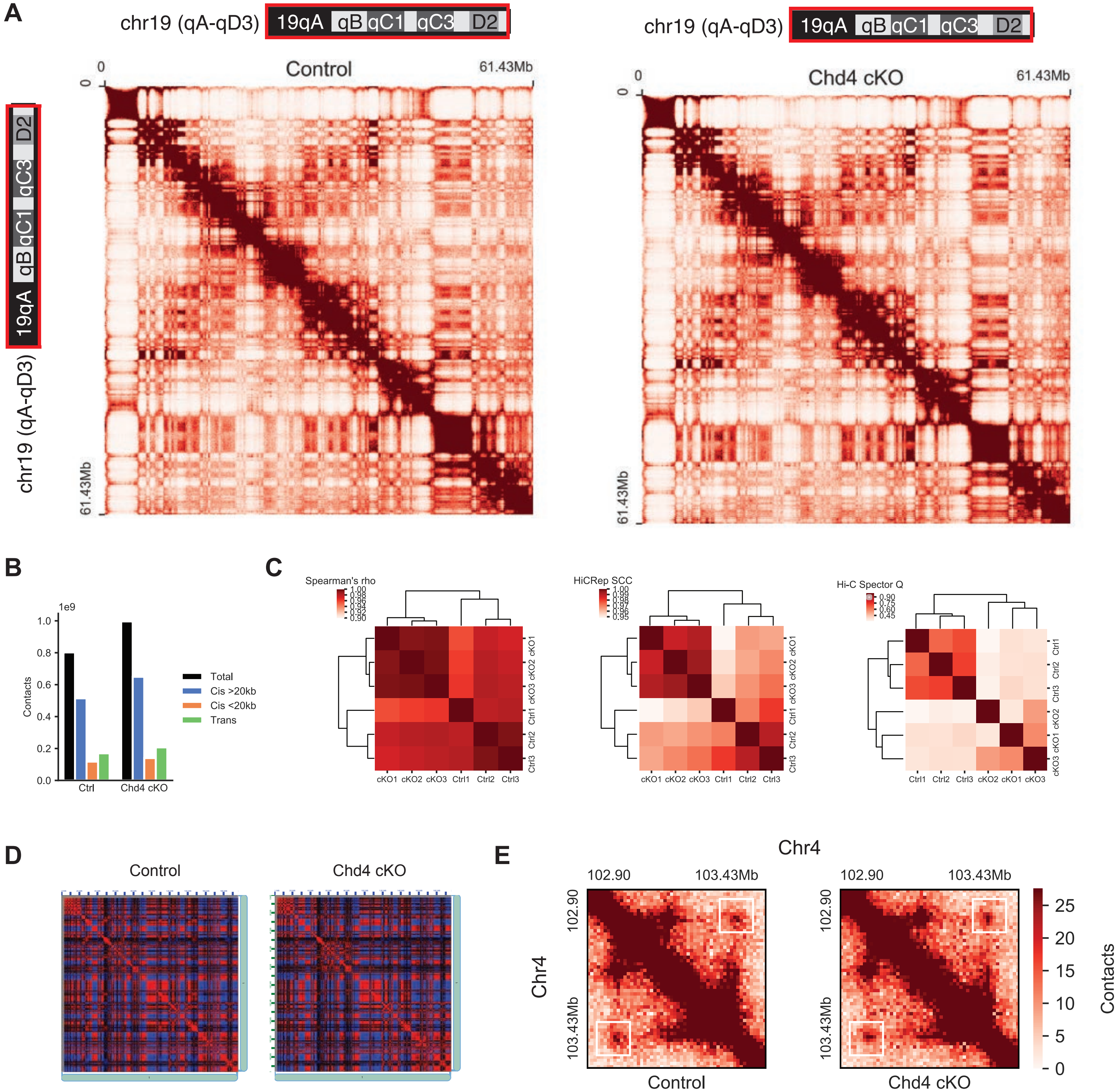

Supp Figure 2

Supplementary Figure 2: Hi-C in the cerebellum of control and Chd4 cKO mice reveals principles of genomic architecture

(A) Hi-C contact matrices of chromosome 19 in the control and Chd4 cKO cerebellum. **(B)** Distribution of pooled Hi-C contacts in the control (ctrl) and Chd4 cKO cerebellum. **(C)** Clustering of Hi-C contacts in 1Mb bins by Spearman's Rho (left), HiCRep (middle), and Hi-C Spector (right) of the three biological replicates from the control and Chd4 cKO cerebellum. **(D)** Juicebox browser snapshot of the Pearson's correlation matrix of chromosome 1. **(E)** Hi-C contact matrices of a region on chromosome 4 depicted small-scale features such as contact domains and genomic loops (highlighted).

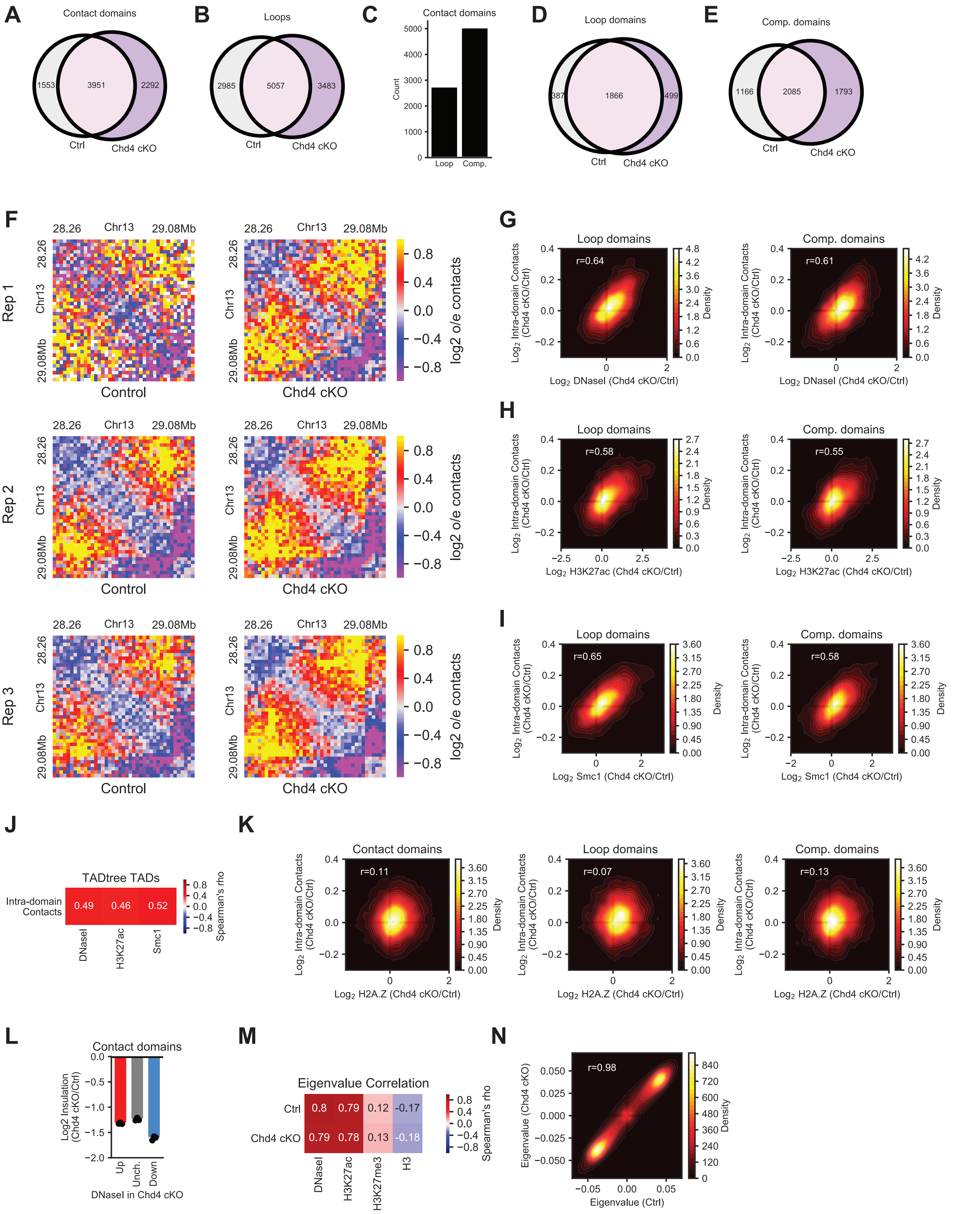

Supp Figure 3

Supplementary Figure 3: Chd4 loss alters interactions within contact domains

(A-B) Overlap of contact domains and loops identified in the control and Chd4 cKO cerebellum. **(C)** Distribution of loop and compartmental domains identified among all contact domains. Chi-squared test. ****p<0.001* **(D-E)** Overlap of loop (D) and compartmental (E) domains identified in the control and Chd4 cKO cerebellum. **(F)** Hi-C contact matrix of a contact domain on chromosome 13 and the flanking region as in Figure 2A from individual biological replicates of the control and Chd4 cKO cerebellum. **(G-I)** Density plots comparing the change in (G) DNaseI, (H) H3K27ac, and (I) Smc1 within a loop (left) or compartmental (right) domain and the change in interactions in that domain between the Chd4 cKO and control cerebellum. Pearson's r, *p<0.001*. **(J)** Correlation of the change in intra-domain contacts (Chd4 cKO/Ctrl) with the change in epigenomic marks (Chd4 cKO/Ctrl) among TADs identified by TADtree. **(K)** Density plots comparing the change in H2A.Z within all (left), loop (middle) or compartmental (right) domain and the change in interactions in that domain between the Chd4 cKO and control cerebellum. Pearson's r, *p<0.001*. **(L)** Change in insulation strength of domain boundaries among domains with increased ($Log_2FC>0.585$), unchanged ($-0.585<Log_2FC<0.585$), and decreased ($-0.585<Log_2FC$) accessibility in the Chd4 cKO cerebellum. Points represent average of domains in individual biological replicates (n=3). **(M)** Correlation of eigenvalue with epigenomic marks in 150kb bins. **(N)** Density plot comparing the eigenvalue of a bin in the control cerebellum to its eigenvalue in the Chd4 cKO cerebellum. Pearson's r, *p<0.001*.

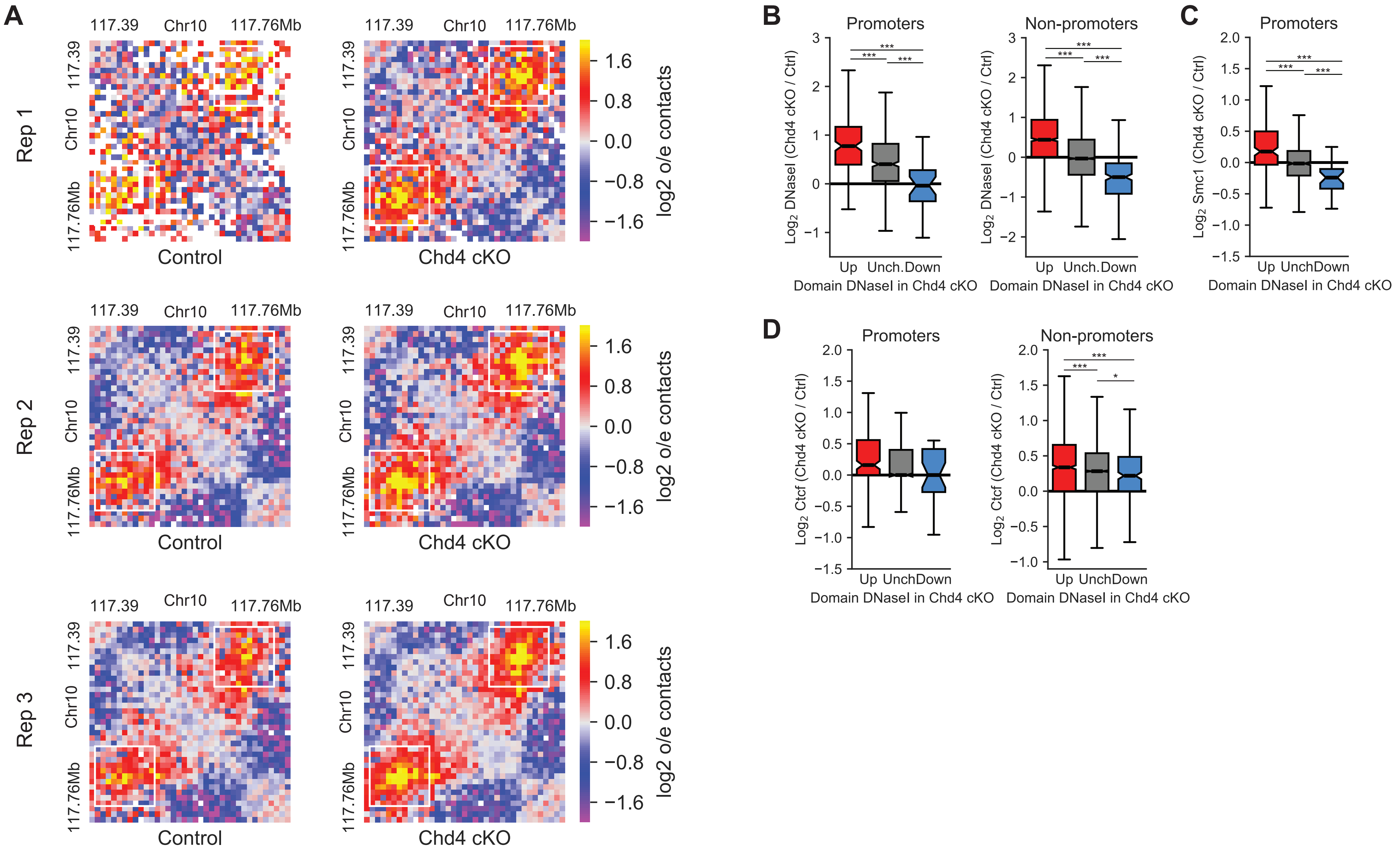

Supp Figure 4

Supplementary Figure 4: Chd4 regulates loop domain boundary loop strength

(A) Hi-C contact matrix of a loop domain on chromosome 10 and the flanking region as in Figure 3A, with a loop domain boundary loop highlighted by a white box, from individual biological replicates of the control and Chd4 cKO cerebellum. **(B-D)** Change in DNaseI (B), Smc1 (C), and Ctcf (D) at promoters and/or non-promoters underlying loop domain boundary loops at domains with increased (n=229 promoters, n=1,343 non-promoters), unchanged (n=711 promoters, n=5,336 non-promoters), or decreased (n=26 promoters, n=241 non-promoters) accessibility in the Chd4 cKO. P-values for all comparisons in this figure were calculated by the two-sided Kruskal-Wallis H-test for independent samples with Dunn's post hoc T-test and corrected for multiple comparisons by the Bonferroni-Hochberg procedure.
p<0.01,*p<0.0001.*

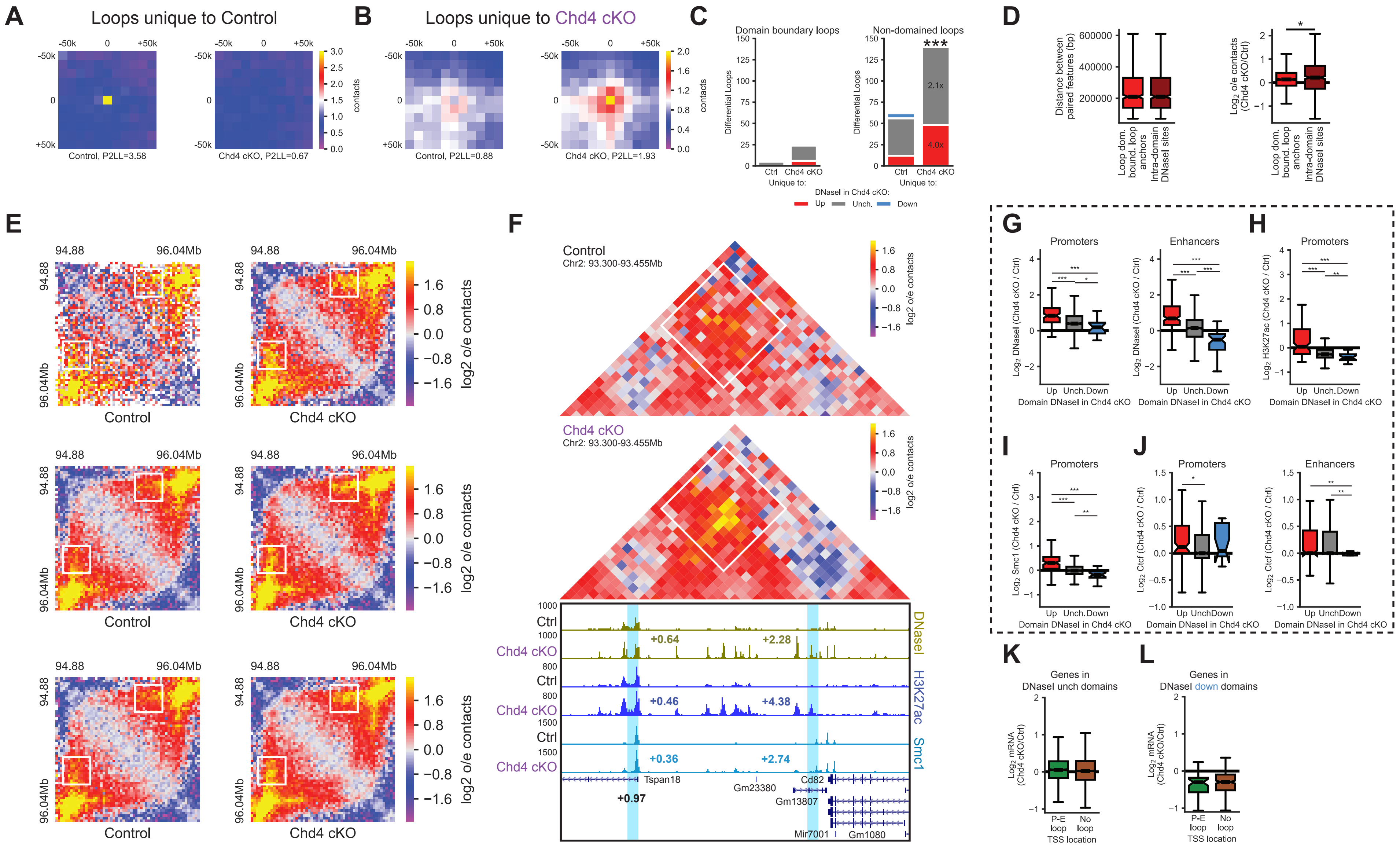

Supp Figure 5

Supplementary Figure 5: Chd4 coordinates intra-domain loop strength and gene expression

(A-B) Aggregate peak analysis of loops unique to the control (A) or Chd4 cKO (B) cerebellum. P2LL, Peak-to-lower-left. **(C)** Location of loops unique to the control or Chd4 cKO cerebellum among domain boundary loops (left) or those not overlapping domain boundaries (right) among domains with increased, unchanged, or decreased accessibility in the Chd4 cKO cerebellum. Inset values indicate the linear fold-change of non-domained loops in domains with increased or unchanged accessibility present in the Chd4 cKO cerebellum compared to the control cerebellum. Chi-square test. ***$p<0.001$ **(D)** (Left) Distance between loop domain boundary loop anchors (n=613) and a distance-controlled random sample of intra-domain DNaseI peak pairs (n=613) and (right) the change in Hi-C contacts at loop domain boundary loops among domains with increased accessibility (n=613) and the random sampling of intra-domain DNaseI peak pairs among domains with increased accessibility upon conditional Chd4 knockout (n=613). * $p = 0.01$ (see methods) **(E)** Hi-C contact matrix of a loop domain on chromosome 4 and the flanking region as in Figure 4A, with an intra-domain enhancer-promoter (E-P) loop highlighted by a white box, from individual biological replicates of the control and Chd4 cKO cerebellum. **(F)** (Top) Hi-C contact matrix of an E-P loop on chromosome 2 and the flanking region, with the loop highlighted by a white box. (Bottom) Genome-browser snapshot of the region corresponding to the Hi-C contact matrix displaying the ChIP-seq profiles of H3K27ac, Smc1, and Ctcf as well as DNaseI-seq from the control and Chd4 cKO cerebellum. Blue denotes the the loop anchors and regions of the insets. Numbers indicate the Log2 change in signal in the Chd4 cKO cerebellum, including that of mRNA for *Tspan18*. **(G-J)** Change in DNaseI (G), H3K27ac (H), Smc1 (I), and Ctcf (J) at promoters and/or enhancers underlying intra-domain E-P loops in domains with increased (n=107 promoters, n=183 enhancers), unchanged (n=795 promoters, n=1,682 enhancers), or decreased (n=19 promoters, n=45 enhancers) accessibility in the Chd4 cKO cerebellum. Two-sided Kruskal-Wallis H-test for independent samples with Dunn's post hoc T-test and corrected for multiple comparisons by the Bonferroni-Hochberg procedure. *$p<0.01$, **$p<0.001$, ***$p<0.0001$. **(K-L)** Change in mRNA of genes at intra-domain E-P loops (green) or underlying no detectable loop (brown) in domains with unchanged (K, n=1,342 at loops, n=11,307 not at loop) or decreased (L, n=50 at loops, n=261 not at loop) accessibility in the Chd4 cKO.

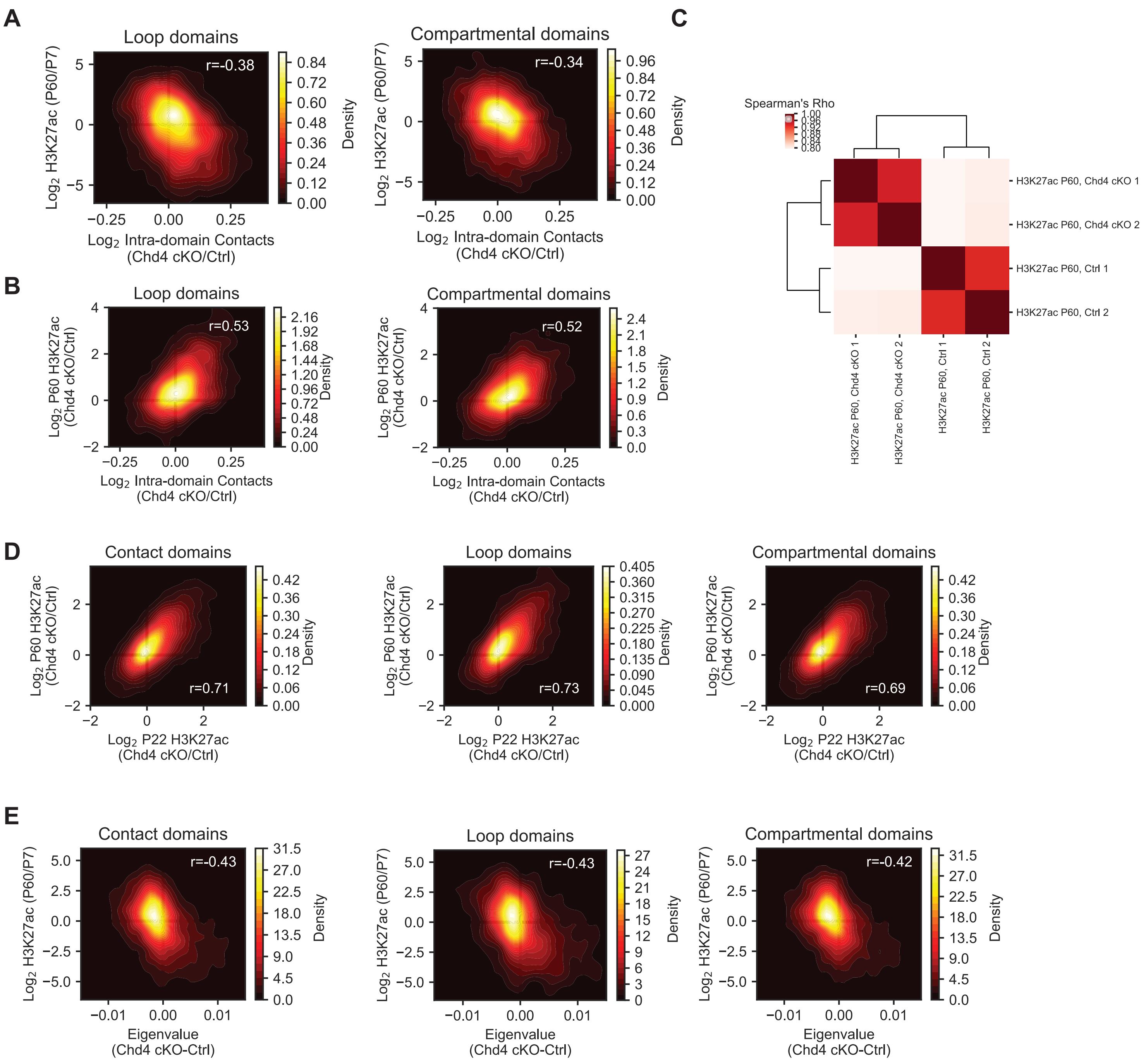

Supp Figure 6

Supplementary Figure 6: Dysregulated contact domains exhibit developmental regulation

(A-B) Density plots comparing the change in loop (A) or compartmental (B) domain contacts between the Chd4 cKO and control cerebellum and the change in H3K27ac in that domain in the cerebellum between P7 to P60. Pearson's r, $p<0.001$. **(C)** Clustering of biological replicates of H3K27ac from the control and Chd4 cKO cerebellum at P60 using Spearman's Rho. **(D)** Density plots comparing the change in H3K27ac at P60 between the control and Chd4 cKO cerebellum to the change in H3K27ac at P22 between the control and Chd4 cKO cerebellum among all (left), loop (middle), or compartmental (right) domains. Pearson's r, $p<0.001$. **(E)** Density plots comparing the change in eigenvalue of all (left), loop (middle) or compartmental (right) domains between the Chd4 cKO and control cerebellum and the change in H3K27ac in that domain in the cerebellum between P7 to P60. Pearson's r, $p<0.001$.
