# Supplementary material for: The chromatin remodeling enzyme Chd4 regulates genome architecture in the mouse brain
Source: Nat Commun. 2020 Jul 9;11:3419. doi: 10.1038/s41467-020-17065-z (PMC7347877; doi:10.1038/s41467-020-17065-z)
Supplement: Supplementary file 2 — Reporting Summary [file 41467_2020_17065_MOESM2_ESM.pdf]

nature research

Corresponding author(s): Azad Bonni, MD, PhD

Last updated by author(s): May 11, 2020

Reporting Summary

Nature Research wishes to improve the reproducibility of the work that we publish. This form provides structure for consistency and transparency in reporting. For further information on Nature Research policies, see Authors & Referees and the Editorial Policy Checklist .

Statistics

For all statistical analyses, confirm that the following items are present in the figure legend, table legend, main text, or Methods section.

n/a	Confirmed	
☐	☒	The exact sample size (*n*) for each experimental group/condition, given as a discrete number and unit of measurement
☐	☒	A statement on whether measurements were taken from distinct samples or whether the same sample was measured repeatedly
☐	☒	The statistical test(s) used AND whether they are one- or two-sided<br>*Only common tests should be described solely by name; describe more complex techniques in the Methods section.*
☒	☐	A description of all covariates tested
☐	☒	A description of any assumptions or corrections, such as tests of normality and adjustment for multiple comparisons
☐	☒	A full description of the statistical parameters including central tendency (e.g. means) or other basic estimates (e.g. regression coefficient) AND variation (e.g. standard deviation) or associated estimates of uncertainty (e.g. confidence intervals)
☐	☒	For null hypothesis testing, the test statistic (e.g. *F*, *t*, *r*) with confidence intervals, effect sizes, degrees of freedom and *P* value noted<br>*Give P values as exact values whenever suitable.*
☒	☐	For Bayesian analysis, information on the choice of priors and Markov chain Monte Carlo settings
☒	☐	For hierarchical and complex designs, identification of the appropriate level for tests and full reporting of outcomes
☐	☒	Estimates of effect sizes (e.g. Cohen's *d*, Pearson's *r*), indicating how they were calculated

Our web collection on statistics for biologists contains articles on many of the points above.

Software and code

Policy information about availability of computer code

Data collection	No software was used for data collection.
Data analysis	For DnaseI- and ChIP-seq: Bowtie2 v2.2.5, Samtools v1.3, Deeptools v2.4.2 (or, on Galaxy v3.3.0.0.0), MACS2 v2.1.1.20160309, Diffbind v2.6.6, DESeq2 v1.20.0, Bedtools v2.25.0.<br>For RNA-seq: Cutadapt v1.16, PRINSEQ v0.20.4, STAR v2.5.3a, Picard Tools v2.19.0, qualimap v2.2.1, RSeQC v2.6.2, HTseq v0.6.1, DESeq2 v1.26.0, DAVID v6.8<br>For Hi-C: HiC-Pro v2.10.0, Juicer v1.5.6, juicer-tools v1.8.9 and v1.9.9, Juicebox v1.9.8, TADTree, 3DChromatinReplicate_QC v1.01<br>General: Jupyter notebook v1.0.0, Pandas v0.24.2, Numpy v1.16.2, Scipy v1.2.1, Matplotlib v2.2.4, Matplotlib-Venn v0.11.5, Seaborn v0.9.0

For manuscripts utilizing custom algorithms or software that are central to the research but not yet described in published literature, software must be made available to editors/reviewers. We strongly encourage code deposition in a community repository (e.g. GitHub). See the Nature Research guidelines for submitting code & software for further information.

Data

Policy information about availability of data

All manuscripts must include a data availability statement. This statement should provide the following information, where applicable:

- Accession codes, unique identifiers, or web links for publicly available datasets
- A list of figures that have associated raw data
- A description of any restrictions on data availability

The data generated in this study are available at GSE138822. Published data used in this study are at the following DOIs: P7 and P60 H3K27ac ChIP-seq at 10.1038/nn.3995 (Frank et al., 2015); P22 Control and Chd4 cKO mRNA-seq and H3K27ac ChIP-seq at 10.1016/j.neuron.2014.05.039 (Yamada et al., 2014); and P22 Control and Chd4 cKO Chd4, H3K27me3, H2A.Z and H3 ChIP-seq at 10.1126/science.aad4225 (Yang et al., 2016). Code for generating figures are available upon request.

Field-specific reporting

Please select the one below that is the best fit for your research. If you are not sure, read the appropriate sections before making your selection.

- [x] Life sciences
- [ ] Behavioural & social sciences
- [ ] Ecological, evolutionary & environmental sciences

For a reference copy of the document with all sections, see nature.com/documents/nr-reporting-summary-flat.pdf

Life sciences study design

All studies must disclose on these points even when the disclosure is negative.

Sample size	Standards of the field (https://www.encodeproject.org/about/experiment-guidelines/) were used to determine the number of biological replicates per condition. For all experiments, 2-3 biological replicates were used per condition.
Data exclusions	No data were excluded from analyses.
Replication	The reproducibility of biological replicates were assessed by standard methods. The clustering of individual biological replicates are reported in the supplemental figures of the manuscript. For all experiments, 2-3 biological replicates were used per condition. All replicates were successful.
Randomization	Sex-matched, littermates were used for all experiments.
Blinding	Experimenters were not blinded to the experimental conditions since there are no subjective measures for the analyses.

Reporting for specific materials, systems and methods

We require information from authors about some types of materials, experimental systems and methods used in many studies. Here, indicate whether each material, system or method listed is relevant to your study. If you are not sure if a list item applies to your research, read the appropriate section before selecting a response.

Materials & experimental systems

n/a	Involved in the study	
	x	Antibodies
x		Eukaryotic cell lines
x		Palaeontology
	x	Animals and other organisms
x		Human research participants
x		Clinical data

Methods

n/a	Involved in the study	
	x	ChIP-seq
x		Flow cytometry
x		MRI-based neuroimaging

Antibodies

Antibodies used	Smc1 5µg/100µL lysate (Bethyl A300-055A), Ctcf 3µg/200µL lysate (Millipore 07-729), H3K27ac 0.1µL/500µL lysate (Abcam ab4729), and H3K4me1 3µL/500µL (Active Motif 39297) antibodies were used in this study.
Validation	All antibodies have been extensively used in other studies.<br>Smc1: Siersbaek et al., Mol Cell 2017 (ChIP-seq)<br>Ctcf: Siersbaek et al., Mol Cell 2017 (ChIP-seq); Yamada et al., Nature 2019 (ChIP-seq)<br>H3K27ac: Siersbaek et al., Mol Cell 2017 (ChIP-seq); Yamada et al., Nature 2019 (ChIP-seq)<br>H3K4me1: Du et al., Nat Comm 2019 (ChIP-seq), Wohlfahrt et al., Nature 2019 (ChIP-qPCR)

Animals and other organisms

Policy information about studies involving animals; ARRIVE guidelines recommended for reporting animal research

Laboratory animals	Male and female Chd4(f/f) and Chd4(f/f); Gabra6-cre/+ mice at P22 and P60 were used in this study. Sex-matched littermates were used for all experiments. Animals were housed under pathogen-free conditions. Experiments were performed in accordance with protocols approved by the Animal Studies Committee at Washington University in St. Louis School of Medicine and National Institutes of Health guidelines.
Wild animals	This study did not involve wild animals.
Field-collected samples	This study did not involve field-collected samples.

Ethics oversight | Experiments were performed in accordance with protocols approved by the Animal Studies Committee at Washington University in St. Louis School of Medicine and National Institutes of Health guidelines.

Note that full information on the approval of the study protocol must also be provided in the manuscript.

ChIP-seq

Data deposition

- [x] Confirm that both raw and final processed data have been deposited in a public database such as GEO.
- [x] Confirm that you have deposited or provided access to graph files (e.g. BED files) for the called peaks.

Data access links
May remain private before publication.

https://www.ncbi.nlm.nih.gov/geo/query/acc.cgi?acc=GSE138822

Files in database submission

P22 DSG Input Ctrl
P22 DSG Input Chd4 cKO
P60 Input Ctrl
P60 Input Chd4 cKO
P22 H3K4me1 Ctrl 1
P22 H3K4me1 Ctrl 2
P22 H3K4me1 Chd4 cKO 1
P22 H3K4me1 Chd4 cKO 2
P60 H3K27ac Ctrl 1
P60 H3K27ac Ctrl 2
P60 H3K27ac Chd4 cKO 1
P60 H3K27ac Chd4 cKO 2
P22 nuclear RNA-seq Ctrl 1
P22 nuclear RNA-seq Ctrl 2
P22 nuclear RNA-seq Ctrl 3
P22 nuclear RNA-seq Ctrl 4
P22 nuclear RNA-seq Chd4 cKO 1
P22 nuclear RNA-seq Chd4 cKO 2
P22 nuclear RNA-seq Chd4 cKO 3
P22 nuclear RNA-seq Chd4 cKO 4
Hi-C Ctrl1
Hi-C Ctrl2
Hi-C Ctrl3
Hi-C Chd4 cKO1
Hi-C Chd4 cKO2
Hi-C Chd4 cKO3
DNaseI-seq Ctrl 1
DNaseI-seq Ctrl 2
DNaseI-seq Chd4 cKO1
DNaseI-seq Chd4 cKO2
Ctcf ChIP-seq Ctrl1
Ctcf ChIP-seq Ctrl2
Ctcf ChIP-seq Chd4 cKO1
Ctcf ChIP-seq Chd4 cKO2
Smc1 ChIP-seq Ctrl1
Smc1 ChIP-seq Ctrl2
Smc1 ChIP-seq Chd4 cKO1
Smc1 ChIP-seq Chd4 cKO2

Genome browser session
(e.g. UCSC)

N/A

Methodology

Replicates | All biological replicates were in high agreement.

Sequencing depth

Sample Platform Read length Single/Paired-end Raw reads
P22 Smc1 Ctrl 1 Illumina HiSeq 2500 50 Single 23240973
P22 Smc1 Ctrl 2 Illumina HiSeq 2500 50 Single 27776746
P22 Smc1 Chd4 cKO 1 Illumina HiSeq 2500 50 Single 23384337
P22 Smc1 Chd4 cKO 2 Illumina HiSeq 2500 50 Single 22571255
P22 nuclear RNA-seq Ctrl 1 Illumina NextSeq 500 37 Paired 100538032
P22 nuclear RNA-seq Ctrl 2 Illumina NextSeq 500 37 Paired 90753329
P22 nuclear RNA-seq Ctrl 3 Illumina NextSeq 500 37 Paired 135637388

P22 nuclear RNA-seq Ctrl 4 Illumina NextSeq 500 37 Paired 79155694
P22 nuclear RNA-seq Chd4 cKO 1 Illumina NextSeq 500 37 Paired 129873359
P22 nuclear RNA-seq Chd4 cKO 2 Illumina NextSeq 500 37 Paired 118066360
P22 nuclear RNA-seq Chd4 cKO 3 Illumina NextSeq 500 37 Paired 105295515
P22 nuclear RNA-seq Chd4 cKO 4 Illumina NextSeq 500 37 Paired 85695887
P22 H3K4me1 Ctrl 1 Illumina NextSeq 500 37 Paired 48029296
P22 H3K4me1 Ctrl 2 Illumina NextSeq 500 37 Paired 40368383
P22 H3K4me1 Chd4 cKO 1 Illumina NextSeq 500 37 Paired 42783915
P22 H3K4me1 Chd4 cKO 2 Illumina NextSeq 500 37 Paired 43447027
P60 H3K27ac Ctrl 1 Illumina NextSeq 500 37 Paired 47176296
P60 H3K27ac Ctrl 2 Illumina NextSeq 500 37 Paired 51835274
P60 H3K27ac Chd4 cKO 1 Illumina NextSeq 500 37 Paired 49913178
P60 H3K27ac Chd4 cKO 2 Illumina NextSeq 500 37 Paired 50055896
P22 Hi-C Ctrl 1 Illumina NextSeq 500 75 Paired 287280942
P22 Hi-C Ctrl 2 Illumina NextSeq 500 75 Paired 1183349373
P22 Hi-C Ctrl 3 Illumina NextSeq 500 75 Paired 1667452346
P22 Hi-C Chd4 cKO 1 Illumina NextSeq 500 75 Paired 364338441
P22 Hi-C Chd4 cKO 2 Illumina NextSeq 500 75 Paired 1005195177
P22 Hi-C Chd4 cKO 3 Illumina NextSeq 500 75 Paired 1695975246
P22 DSG Input Ctrl Illumina HiSeq 2500 50 Single 26677624
P22 DSG Input Chd4 cKO Illumina HiSeq 2500 50 Single 19956203
P60 Input Ctrl Illumina NextSeq 500 37 Paired 46638330
P60 Input Chd4 cKO Illumina NextSeq 500 37 Paired 47176296
P22 Ctcf Ctrl 1 Illumina NextSeq 500 36 Paired 26304363
P22 Ctcf Ctrl 2 Illumina NextSeq 500 36 Paired 25646604
P22 Ctcf Chd4 cKO 1 Illumina NextSeq 500 36 Paired 25267961
P22 Ctcf Chd4 cKO 2 Illumina NextSeq 500 36 Paired 28582219
P22 DNaseI Ctrl 1 Illumina HiSeq 2500 50 Single 134358116
P22 DNaseI Ctrl 2 Illumina HiSeq 2500 50 Single 125296697
P22 DNaseI Chd4 cKO 1 Illumina HiSeq 2500 50 Single 116572781
P22 DNaseI Chd4 cKO 2 Illumina HiSeq 2500 50 Single 140279257

Antibodies

Smc1 (Bethyl A300-055A), Ctcf (Millipore 07-729), H3K27ac (Abcam ab4729), and H3K4me1 (Active Motif 39297) were used in this study.

Peak calling parameters

DNaseI-seq peaks were called using MACS2 (v2.1.1.20160309) at a q-value of less than 0.01 (-q 0.01) without model building (--nomodel), an extension of 200bp (--extsize 200), and a shift of -100bp (--shift -100) (Zhang et al., 2008). Peaks from control and Chd4 cKO were then merged and called as significantly different using Diffbind (v2.6.6) running DESeq2 (v1.20.0) (Love et al., 2014; Ross-innes et al., 2012). Chd4 and H3K4me1 ChIP-seq peaks were called using MACS2 (v2.1.1.20160309) using the broad settings (--broad) at a q-value of less than 0.05 (-q 0.05 –-broad-cutoff 0.05) without model building (--nomodel) and an extension of 300bp (--extsize 300).

Data quality

Number of peaks called:
DNaseI-seq, 121790 peaks
H3K4me1, 84425 peaks

Software

For DnaseI- and ChIP-seq: Bowtie2 v2.2.5, Samtools v1.3, Deeptools v2.4.2 (or, on Galaxy v3.3.0.0.0), MACS2 v2.1.1.20160309, Diffbind
v2.6.6, DESeq2 v1.20.0, Bedtools v2.25.0.
For RNA-seq: Cutadapt v***, PRINSEQ v0.20.4, STAR v2.5.3a, Picard Tools v2.19.0, qualimap v2.2.1, RSeQC
v2.6.2, HTseq v0.6.1, DESeq2 v1.26.0, DAVID v6.8
For Hi-C: HiC-Pro v2.10.0, Juicer v1.5.6, juicer-tools v1.8.9 and v1.9.9, Juicebox v1.9.8, TADTree, 3DChromatinReplicate_QC v1.01
General: Jupyter notebook v1.0.0, Pandas v0.24.2, Numpy v1.16.2, Scipy v1.2.1, Matplotlib v2.2.4, Matplotlib-Venn v0.11.5, Seaborn v0.9.0
